# Leveraging Knowledge of Traditional Italian Maize Landrace Diversity to Identify Signals of Local Adaptation

**DOI:** 10.1111/eva.70186

**Published:** 2026-02-13

**Authors:** Alessandra Lezzi, Lorenzo Stagnati, Leonardo Caproni, Matteo Dell'Acqua, Matteo Busconi, Alessandra Lanubile, Adriano Marocco

**Affiliations:** ^1^ Department of Sustainable Crop Production Università Cattolica Del Sacro Cuore Piacenza Italy; ^2^ Research Centre for Biodiversity and Ancient DNA Università Cattolica Del Sacro Cuore Piacenza Italy; ^3^ Institute of Plant Sciences Scuola Superiore Sant'anna Pisa Italy

**Keywords:** climate adaptation, landraces, landscape genomics, partial redundancy analysis, plant genetic resources, *Zea mays* L.

## Abstract

Climate change is the greatest challenge to modern agriculture. It significantly impacts agricultural systems through an increased frequency and intensity of extreme environmental events. Maize, a vital crop for global food security, is particularly vulnerable to these changes, highlighting the urgent need to develop resilient varieties. This study aims to identify significant genes for adaptation to environmental conditions in 140 individuals derived from 28 Italian maize landraces using a landscape genomics approach to support the development of resilient maize genotypes. Landraces were genotyped using genotyping‐by‐sequencing, and the resulting genetic matrix was used to characterize the collection's diversity. Population genetic studies were conducted to investigate the genetic diversity and structure of the collection. Partial redundancy analysis (pRDA) was subsequently employed to analyze the relationship between climate variables and genetic variation of the materials. Among the 12 ancestral populations identified, both well‐defined populations and highly admixed groups were observed. This degree of admixture was reflected in the clustering analysis and principal component analysis (PCA), although clear differentiation of individual populations was still apparent. pRDA revealed that 30% of the genetic variance in the collection was explained together by climate (45%), geography (11%), and genetic structure (31%). Three potential genomic signals of adaptation were identified in relation to the environmental variability across the sampling sites. The results highlight significant intra‐landrace variability within the examined germplasm and reveal unique landraces tied to ancestral lineages. Notably, we identify distinct genetic markers strongly correlated with environmental factors. This discovery opens new avenues for potential genetic improvement in maize cultivation. Landraces preserve vital traits for the adaptation of maize to environmental stresses, thereby serving as key sources for breeding programs aimed at improving stress tolerance and yield stability under climate change.

## 1 Introduction

The cultivation of maize ( *Zea mays* L. subsp. *mays*) holds fundamental importance in worldwide cereal production, serving both human and animal consumption. The crop is a global leader in terms of annual production, surpassing one billion tons (Erenstein et al. 2022) and ranking second in cultivated area after wheat (Erenstein et al. 2022).

Current climate change scenarios predict temperature increases and changes in the frequency and severity of extreme events, including heavy rainfall, floods, droughts, and heatwaves, as well as shifts in precipitation patterns—encompassing the intensity, duration, and frequency of abnormal heat or cold periods (Kogo et al. 2019; Mereu et al. 2021). Climate change is expected to significantly impact Italian cereal production, particularly maize, in the mid to long term (Mereu et al. 2021). The primary impact on maize yield arises from rising temperatures, which accelerate the growth cycle and reduce biomass. In Italy, approximately 8% of the total cultivable land is dedicated to maize (Istat Statistics 2024). It is predicted that irrigation needs will decrease in the South of Italy due to tropicalization, while Central and Northern regions will suffer from prolonged drought. In contrast, the projected impact from future CO_2_ levels is minimal (Mereu et al. 2021).

Under this scenario, it is essential to develop hybrids better adapted to changing environmental conditions, facilitated by a broader genetic base (Goodman 2005; Călugăr et al. 2024). Maize landraces—adapted to diverse agroecological conditions—can offer invaluable diversity for research and future genetic improvement (Prasanna 2012).

Italy, considered a secondary centre of maize diversification, is particularly rich in landraces empirically selected over the centuries by farmers for better adaptation to specific environments, flowering behaviour, yield, taste, and traits related to both utilization and tolerance to biotic and abiotic stresses (Louette and Smale 2000; Pressoir and Berthaud 2004; Brandolini and Brandolini 2009; Sangiorgio et al. 2021). High genetic variability and tolerance to natural and anthropogenic environments make landraces invaluable genetic resources used by breeders to harness useful genes and alleles for stress tolerance and resistance (Brush 1995; Hartings et al. 2008; Newton et al. 2009; Lanzanova et al. 2019; Di Pasquale et al. 2024).

A popular method employed to identify and utilize the genetic variation present in local populations involves determining the extent to which their genomes are shaped by artificial and natural selection. This approach entails the search for adaptive loci within their genomes (Joost et al. 2007). Investigating the relationship between adaptive genetic signatures in genomes and environmental heterogeneity among natural populations is the focus of the relatively new field of landscape genomics, which has become an important tool in current research (Li et al. 2017).

In research on cultivated cereals, landscape genomics has demonstrated its potential in detecting loci putatively related to adaptation. Dell'Acqua et al. (2014) investigated local adaptation in *Brachypodium distachyon* (L.) P. Beauv., identifying 15 genes significantly linked to environmental adaptation in accessions from Turkey. Brunazzi et al. (2018) applied this approach to assess environmental adaptation in natural populations of *Triticum urartu* Thumanjan ex Gandilyan, 1972, resulting in the identification of several significant loci. In terms of agricultural species, the identification of putative adaptive loci in *Hordeum vulgare* L. was first reported by Abebe et al. (2015). Caproni et al. (2023) subsequently identified adaptive loci in the same crop. Rhoné et al. (2020) employed landscape genomics to investigate the impact of climate change on pearl millet cultivation in West Africa, identifying future vulnerable areas where landraces could contribute to mitigating climate change effects. Two recent studies have employed landscape genomics to detect associations between molecular markers and environmental variables in maize. In particular, Westengen et al. (2012) detected adaptive loci responsive to precipitation and maximum temperature in African maize landraces using association analysis. More recently, Tamang et al. (2024) assessed the agronomic and climatic diversity and adaptation of maize landraces in Bhutan, identifying several quantitative trait nucleotides (QTNs) significantly associated with bioclimatic variables. These QTNs corresponded to genomic regions previously identified for agronomic traits such as flowering time, inflorescence, seed composition, and vegetative traits.

In this study, we performed the genetic characterization of 140 individuals derived from 28 representative landraces from Northern‐Central Italy, with five individuals sampled per landrace. A landscape genomic approach using partial redundancy analysis (pRDA) was applied to associate genetic markers with bioclimatic variables, aiming to identify candidate genes useful for developing more climate‐resilient maize varieties.

## 2 Methods

### 2.1 Germplasm and Field Management

A total of 28 traditional maize landraces from Northern‐Central Italy were retrieved from five different germplasm collections: two landraces from Regional Agricultural Institute (Institut Agricole Régional, La Rochère, Aosta); ten from the Council for Agricultural Research and Economics, Research Centre for Cereal and Industrial Crops (CREA‐CI, Bergamo); five from Regional Agency for Development and Innovation in the Agricultural and Forestry Sector (ARSIA, Firenze); seven from the Department of Earth and Environmental Sciences of Università degli Studi di Pavia (UNIPV, Pavia); and four from the Department of Sustainable Crop Production of Università Cattolica del Sacro Cuore (UCSC, Piacenza) (Table S1).

Surveyed landraces represent the most important traditional materials for specific locations and, in some cases, are still cultivated. The landraces were selected to encompass seven distinct regions in Northern‐Central Italy: five from Emilia‐Romagna; five from Toscana; seven from Lombardia; six from Trentino‐Alto Adige; two from both Valle d'Aosta and Veneto; and one from Friuli‐Venezia Giulia. These landraces account for wide phenotypic variability, as derived from field observations described in previous studies (Table S1).

**FIGURE 1:**
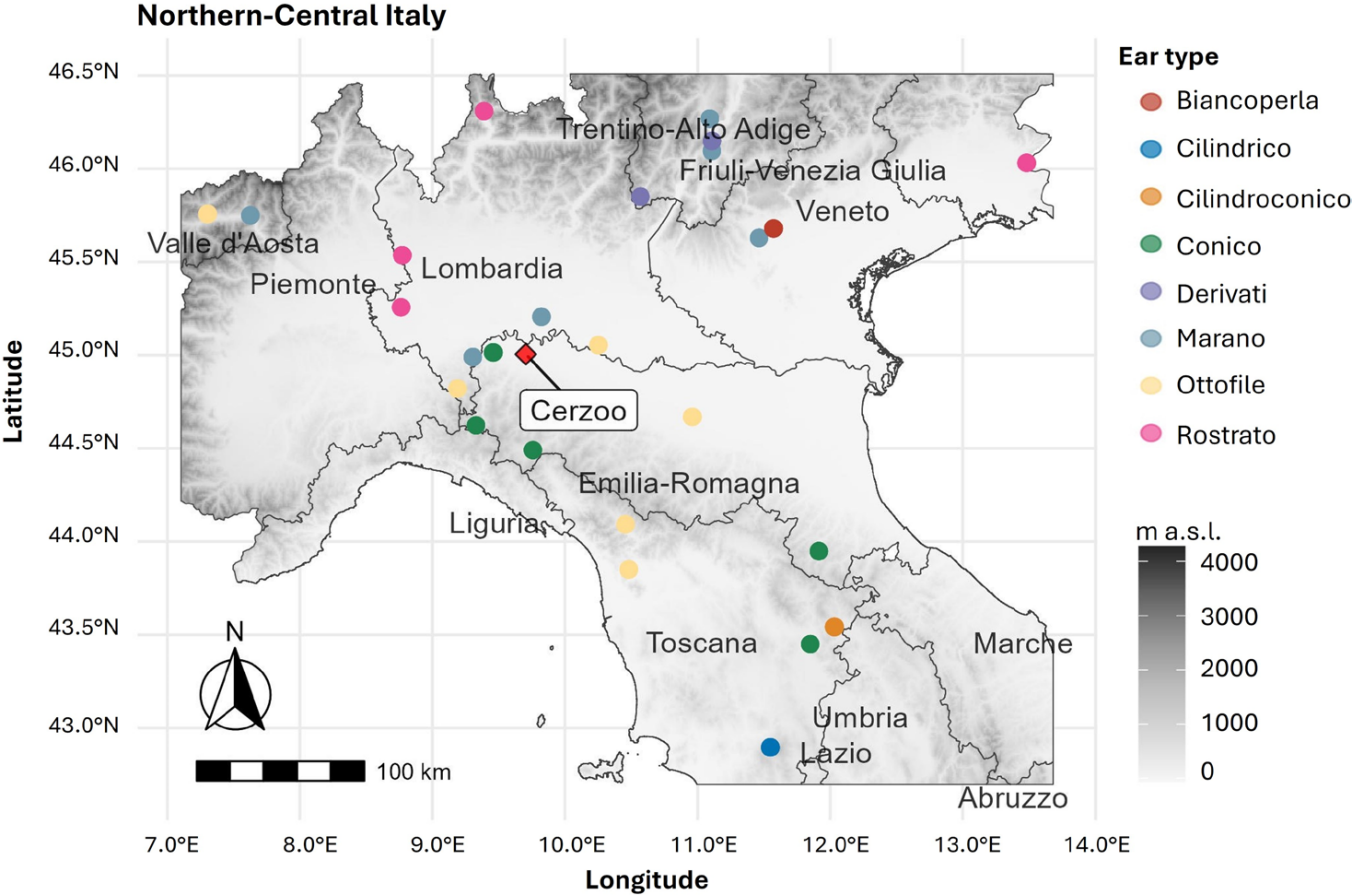
Geographic distribution of the 28 maize landrace collection. Landraces are divided by colors according to their racial complex group as identified by Brandolini and Brandolini (2009). Longitude and latitude values in WGS84 degrees are reported on the *x*‐axis and *y*‐axis, respectively. The sampling area is reported in shades of grey representing altitude according to the bar on the side (m above sea level).

The landraces analyzed in this study belong to seven of the nine racial complexes identified by Brandolini and Brandolini (2009). Specifically, one variety belongs to the “Biancoperla” complex (pearl white flints), characterized by large cylindrical ears and white kernels with a pearly appearance; one variety to the “Cilindrico” complex (Cylindrical flints), which includes ancient long‐eared landraces; and one variety to the “Cilindroconico” complex, an intermediate group between the Cylindrical and Conical flints, comprising landraces with conical ears. The “Ottofile” complex is represented in our collection by six landraces with eight rows of flint kernels. From the introgression of the latter group, “Ottofile”, with other varieties originated the “Derivati” group, characterized by 10–12 rowed derived ears and two sampled landraces. Four landraces belong to the “Rostrato” complex, characterized by ears with beaked tips and deeply dented kernels; five local varieties are assigned to the “Conico” complex (Conical flints), which encompasses landraces bearing conical or subconical ears with medium‐sized, isodiametric, and thick kernels. The large conical ear is considered an adaptation to rainfed environments. Last, six landraces correspond to the “Marano” type, classified by Brandolini and Brandolini (2009) within the Microsperma flints complex and characterized by sub‐cylindrical ears with small and hard kernels, particularly suited for food preparation. Detailed information on individual landrace names, collection sites, and geographic coordinates is provided in Table S1. Figure 1 presents the geographic distribution of the landraces (Brandolini and Brandolini 2006; Ardenghi et al. 2018; Lezzi et al. 2023).

Landraces were cultivated at Centro di Ricerche per la Zootecnia e l'Ambiente (CERZOO) experimental farm (45°0.303960′ N, 9°42.252360′ E, San Bonico, Piacenza, Italy). Multiple plots were assigned to each genotype, with a total of four plots per landrace. Each plot consisted of a single row of plants, 6 m in length, with rows spaced 80 cm apart and a 1 m aisle along the edges. A total of 25 seeds were sown for each row. The field was managed in accordance with standard agricultural practices for maize nursery cultivation. Weed control was conducted with pre‐emergence herbicide (AdengoXtra, Bayer Crop Science, 0.44 L/ha) and additional manual weeding if required. Irrigation was performed by overhead systems.

### 2.2 Plant Material and DNA Extraction

Leaf samples were collected at the V5 stage. In brief, the apical 5–10 cm of the youngest visible leaf in the cone was sampled from five different individuals for each landrace, resulting in a total of 140 samples.

Genomic DNA was extracted with the GenElute Plant Genomic DNA Miniprep Kit (Merck Life Science s.r.l., Darmstadt, Germany) following the manufacturer's instructions, with the minor modification of adding 5% w/v Polyvinylpyrrolidone (PVP) during the lysis step to enhance the removal of polyphenols (Stagnati et al. 2021). DNA quality and concentration were evaluated using a NanoPhotometer N80 (Implen GmbH, München, Germany) and visualized on 1% agarose gel electrophoresis stained with Eurosafe nucleic acid stain (EuroClone S.p.A, Pero (MI), Italy).

### 2.3 Sequencing and Bioinformatic Analysis

Genotyping was performed at Biodiversa s.r.l. Servizi NGS (Treviso, Italy) with a genotyping by sequencing approach (GBS) (Elshire et al. 2011; Dell'Acqua et al. 2014; Abebe et al. 2015) using *Eco*RI and *Msp*I restriction enzymes. Fragments were sequenced on an Illumina NovaSeq S4 platform (Illumina, California, USA).

Raw reads were processed using the *process_radtags* utility in Stacks v2.0 (Catchen et al. 2011). Quality was assessed with the FastQC tool (v 0.11.5, Andrews 2010) and only bases with a Phred Score > 30 were retained for further analysis. The resulting clean reads were mapped to version 5 of the B73 maize reference genome (GCA_902167145, Harper et al. 2016) using the Burrows–Wheeler Aligner based on the Maximal Exact Match algorithm (BWA‐MEM), with default parameters except for the “‐M” flag. The alignment file was then sorted and indexed using SAMtools (Danecek et al. 2021). Quality control analysis was performed using the FastQC tool (v 0.11.5, Andrews 2010) with all the default parameters. Variant detection was conducted using the *ref_map.pl* utility from Stacks with default parameters (Catchen et al. 2011). The distribution of unfiltered SNPs across the genome was plotted using the CMplot package in R (v.4.1.3) (Yin et al. 2021; R Core Team 2022).

The final set of SNPs employed for downstream analyses was restricted to variants with a minor allele frequency (MAF) > 0.05 and missingness > 0.95 (Abebe et al. 2015; Stagnati et al. 2019, 2020; Caproni et al. 2023).

### 2.4 Genetic Features and Diversity of the Collection

The pairwise linkage disequilibrium (LD) was assessed from quality‐filtered SNPs using the *r* ^2^ metrics implemented in the LDheatmap R package (Shin et al. 2006). The LD decay relative to physical distance was modelled using the Hill and Weir equation (Hill and Weir 1988). An *r* ^2^ threshold of 0.3 was used to estimate the LD decay distance for each chromosome (Caproni et al. 2023). The genetic structure and diversity of maize varieties were evaluated using a set of SNP markers pruned by the LD at a threshold of *r* ^2^ = 0.5. LD filtering was conducted using PLINK's (v.1.9, Chang et al. 2015)—*indep‐pairwise* function (Purcell et al. 2007), which requires specification of three parameters: a window size for variant counting, the number of variants by which the window is shifted at each step, and a pairwise correlation threshold (*r* ^2^). Based on the literature, values selected for each parameter were 150, 5, and 0.5, respectively (Calus and Vandenplas 2018; Caproni et al. 2023).

Complete linkage agglomerative clustering, based on pairwise identity‐by‐state (IBS) distance, was performed using PLINK with the default settings. Pairwise kinship matrix value similarities among samples were plotted with the *hclust()* function in R.

The genetic structure and diversity of maize landraces were further investigated through principal component analysis (PCA) (Greenacre et al. 2022; Lezzi et al. 2023) using PLINK (v.1.9, Chang et al. 2015). The results were visualized in R. The population structure was evaluated using ADMIXTURE v.1.3.0 (Alexander et al. 2020), with the number of clusters (*K*) ranging from 2 to 30. To assess the quality of the clustering and infer the most likely *K*‐value, the cross‐validation error was estimated for each *K*‐value. The results of the ADMIXTURE analysis were visualized using the *Pophelper* package in R (Francis 2017). A dendrogram was constructed using the UPGMA algorithm in the *ape* R package to depict the relationships among individuals, with 100 bootstrap replicates to assess branch support (Paradis and Schliep 2019; R Core Team 2022).

The inbreeding coefficient (F), the Hardy–Weinberg equilibrium, and the fixation index per site (F_ST_, Weir and Cockerham 1984) were computed in VCFtools (Danecek et al. 2011) using the—*het*, —*hardy*, and—*weir‐fst‐pop* functions, respectively (Dominguez et al. 2024). The corresponding graphs were plotted with the *ggplot2* package in R (Wickham 2014).

### 2.5 Spatial and Climatic Characterization of the Sampling Locations

The GPS coordinates of the sampling locations were projected onto the map of Northern‐Central Italy using the raster package in R (Hijmans 2025). Climate data of the study area for the period 1970–2000 were obtained from the WorldClim database (version 2) (Fick and Hijmans 2017) at a spatial resolution of 2.5 arc‐minutes. The dataset consisted of 19 bioclimatic variables derived from monthly temperature and rainfall values. These variables, normally used for species distribution modelling approaches, provide biologically meaningful information (Fick and Hijmans 2017). Bioclimatic variables describe annual trends, seasonality, and extreme or limiting environmental factors, often expressed as quarterly values (Fick and Hijmans 2017). Table S2 reports each climate variable used in this study and the respective code. Multi‐collinearity among bioclimatic variables was assessed using the *ensemble.VIF()* function in the BiodiversityR package in R (Kindt and Coe 2005). Variables with a variance inflation factor below 10 were retained for further analysis (Woldeyohannes et al. 2022).

To explore the environmental heterogeneity underlying the studied landraces, we performed PCA using altitude and the non‐collinear bioclimatic variables. Prior to analysis, all variables were centered and scaled to unit variance to account for differences. PCA was conducted using the *prcomp()* function in the *stats* R package (R Core Team 2022), with landraces as operational taxonomic units (OTUs). The proportion of variance explained by each principal component was calculated to assess the contribution of individual axes to the overall environmental variation. To aid interpretation, the loadings of altitude and bioclimatic variables were projected as vectors onto the PCA plot, indicating their relative contribution and direction of influence.

### 2.6 Identification of Genetic Signatures of Adaptation Across the Climatic Landscape

The factors influencing the differentiation of maize landraces across Northern‐Central Italy were determined using pRDA. The set of LD‐pruned SNPs (*r* ^2^ = 0.5) served as the response variables, while environmental factors, geographic coordinates, and genetic structure were used as three independent sets of explanatory variables. The proportion of genetic variance attributable to each set of explanatory variables was estimated by sequentially removing the influence of other sets of variables. Environmental factors were represented by non‐collinear historical bioclimatic variables at the sampling sites. Geographic factors were represented by the latitude and longitude of the landrace sampling points of origin, and the genetic structure was described by the first three principal components derived from the PCA conducted using the same set of LD‐pruned markers (Capblancq and Forester 2021).

Partial redundancy analysis was applied to investigate genotype–environment associations, using bioclimatic data as explanatory variables while accounting for genetic structure represented by PCA. The multivariate approach proposed by Capblancq et al. (2018) was employed to identify associated loci and detect genomic signatures of adaptation. The identification of potential adaptive loci was performed using the vegan R package, and outliers were detected with the *rdadapt()* function, following the filtering, imputation, and interpretation procedures outlined by Capblancq and Forester (2021).

A gradient forest (GF) machine‐learning regression tree‐based algorithm was implemented with the *gradientForest* package in R (Ellis et al. 2012) to determine the environmental variables that most effectively explain the genetic variation of maize landraces across the study area. The GF assesses the predictive power of each bioclimatic variable in explaining allele changes and builds a function to predict the expected genetic combinations of the potential climate adaptive loci (i.e., those that are well predicted by the GF model, characterized by positive *r* ^*2*^ values). Besides the bioclimatic variables, the GF model was also trained using Moran's Eigenvector Maps (MEMs), derived using the *dbmem()* function from the adespatial package in R (Dray et al. 2012). MEMs are uncorrelated eigenvectors determined from the pairwise spatial weighting matrix of the geographic coordinates that describe the spatial distribution of the sampling points. To build the GF function, a forest of 500 trees was constructed for each SNP as the response variable, using non‐collinear bioclimatic variables and MEMs as predictors.

In order to identify outlier loci emerging from both pRDA and GF analyses, we compared the *p*‐values of individual SNPs estimated via pRDA with the *r* ^*2*^ values of the same set of SNPs (Rhoné et al. 2020), applying a Bonferroni threshold with a nominal *p*‐value of 0.05 and selecting *r* ^*2*^ values above the 95th percentile of the distribution, respectively.

### 2.7 Identification of Candidate Genes

Once adaptive loci were identified through outlier detection, their genomic positions were examined on the B73 maize genome (version 5, GCA_902167145) using the JBrowse genome browser (Goodstein et al. 2012). For SNPs located within gene models, gene functional annotation was derived using Phytozome 14 (Goodstein et al. 2012), which provides a list of best‐hit orthologs based on the amino acid sequences. After the identification of each gene, additional information was also derived using the MaizeGDB database (Harper et al. 2016) and relevant literature. The same approach was applied to analyse genes within the same LD window, centred on the positions of each significant SNP. The LD window is chromosome‐specific and was calculated as described in Section 2.4. Finally, the roles of the potential genes were discussed based on their functions, acknowledging that the identified genes are not necessarily causal.

## 3 Results

### 3.1 Genetic Characterization of the Landraces

GBS sequencing of the 140 maize individuals yielded 1,437,328 raw variants evenly distributed across all maize chromosomes, with no uncovered regions and few low SNP‐density areas (Figure S1).

Quality, missingness, and MAF filtering of the dataset resulted in 6002 variants. The LD estimation decay revealed that, on average, it decayed within 64.27 Kb, with a variation across the ten maize chromosomes (Table S3 and Figure S2). Following LD pruning, the dataset was reduced to 2880 SNP markers, providing an unbiased representation of the diversity existing in the collection. Genetic similarity analysis revealed that landraces were well defined and distinguishable from one another, with some exceptions where groups of more closely related materials were detectable (Figure S3).

Clustering analysis (Figure S4) indicated all varieties to be distinct from each other, with each displaying considerable internal variability. Among the 28 local varieties, Ottofile Mantovano was the most distinct. The dendrogram was subsequently split into two large groups. The first group included varieties such as Biancoperla, Dente di Cavallo Bianco, Scagliolo del Ticino, Rostrato di Mortara, and Rostrato della Valchiavenna, with Biancoperla determined to be the most genetically distant from the others. The second group included Entrebin, while the remaining landraces were difficult to classify, with the possible exception of Marano Gardolo, Marano, Quarantino Cremonese, Nostrano Storo, and Locale Zambana, which could be considered part of the broader “Marano” or “Marano‐related” landrace groups (Brandolini and Brandolini 2009).

**FIGURE 2:**
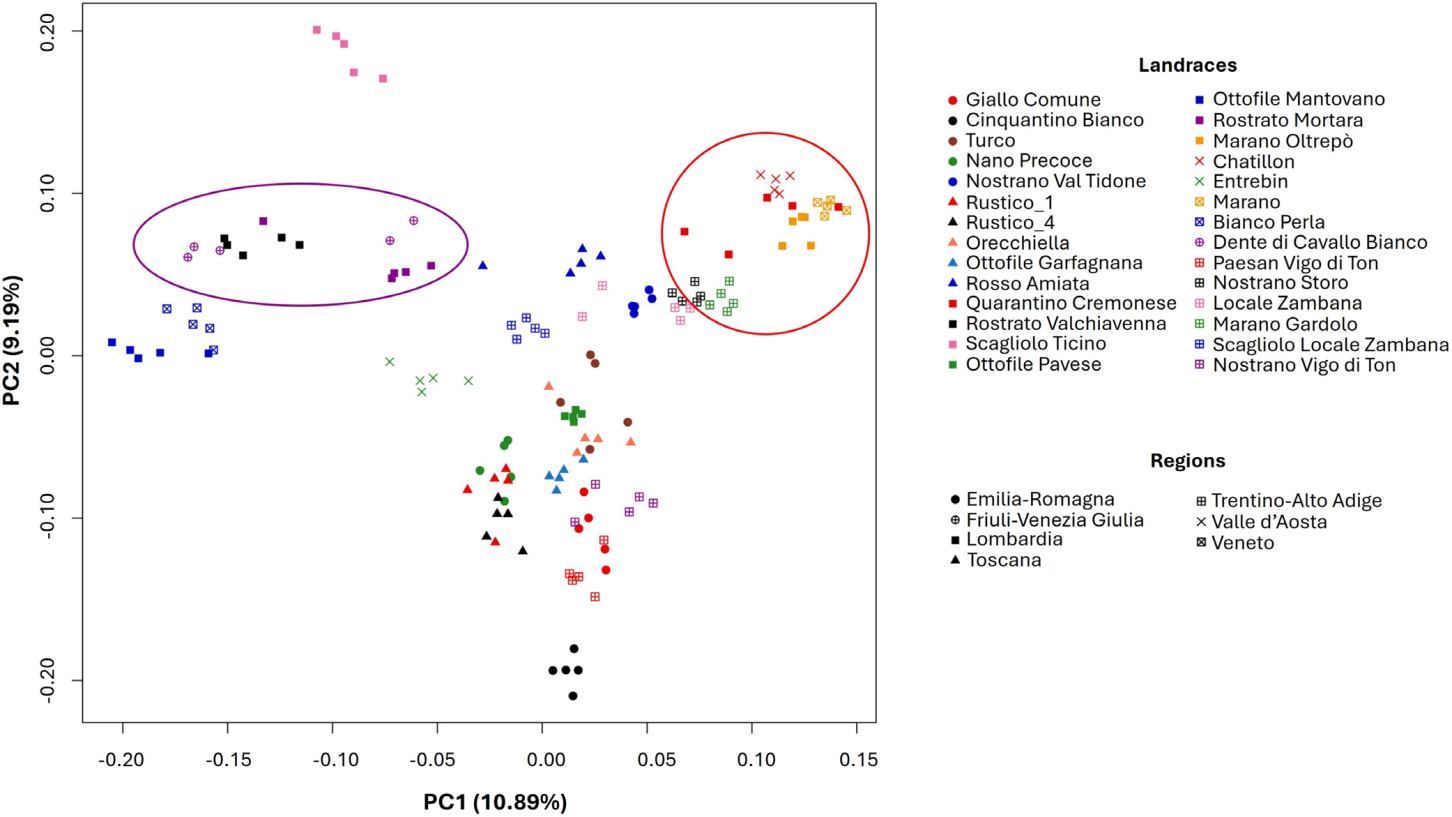
Principal Component Analysis (PCA) score plot based on the LD‐pruned SNP dataset of the 140 samples. The “Rostrati” (purple circle) and “Marani” (red circle) groups are highlighted.

PCA explained 20% of the total genetic variability, with the first two principal components accounting for 10.89% and 9.19%, respectively (Figure 2; Figure S5). Although populations exhibited clear differentiation, a distinct grouping of specific varietal types was not always evident. Landraces such as Cinquantino Bianco and Scagliolo del Ticino displayed tightly clustered individuals clearly grouped together, while other varieties did not. Three notable clusters could be identified: a cluster of Rostrati‐type maize (highlighted in purple in Figure 2) including Dente di Cavallo Bianco (Friuli‐Venezia Giulia), Rostrato della Valchiavenna, and Rostrato di Mortara (Lombardia); a cluster of “Marano” or “Marano‐related” landraces (highlighted in red in Figure 2) including Quarantino Cremonese and Marano Oltrepo (Lombardia), Châtillon (Valle d'Aosta), Marano (Veneto), Nostrano di Storo, and Marano Gardolo (Trentino‐Alto Adige); and a larger cluster (bottom of Figure 2) including landraces from Emilia‐Romagna, Toscana, and Trentino‐Alto Adige. No clear genetic differentiation was observed among landraces based on geographic region.

Admixture analysis indicated that the optimal number of ancestral clusters was *K* = 12 (cross‐validation error = 0.579; Figures S6 and S7C). Ten ancestral groups corresponded to single landraces (Nostrano Val Tidone, Marano Oltrepò, Ottofile Mantovano, Scagliolo Ticino, Rosso Amiata, Nostrano di Storo, Châtillon, Entrebin, Biancoperla, and Marano). Moreover, shared ancestry was observed for Cinquantino Bianco–Paesan Vigo di Ton (Figure S7C, cerulean bars) and Rustico 4–Ottofile Pavese (Figure S7C, green bars).

A more detailed examination of the admixture results revealed three additional groups: the first included Giallo Comune and Rustico 1, related to the Rustico 4–Ottofile Pavese ancestry; the second included Marano Gardolo and Locale Zambana, related to Nostrano di Storo; and a third included landraces belonging to the Rostrati‐type (Dente di Cavallo Bianco, Rostrato di Mortara, and Rostrato della Valchiavenna) as well as Biancoperla. This is consistent with the PCA results. The remaining landraces displayed composite genetic backgrounds resulting from multiple ancestral contributions. Similar to the PCA results, no clear genetic differentiation was observed based on geographic origin.

Although *K* = 12 provided the best fit to the data, clustering solutions were also explored at *K* = 9 (cross‐validation error = 0.586, Figures S6 and S7A), *K* = 11 (cross‐validation error = 0.583, Figures S6 and S7B), and *K* = 13 (cross‐validation error = 0.601, Figures S6 and S7D). The key patterns observed at *K* = 12 were largely maintained across these alternative partitions. At *K* = 9 and *K* = 11, several landraces were predominantly assigned to single ancestral populations, such as those from Emilia‐Romagna (Cinquantino Bianco, Nostrano Val Tidone); Friuli‐Venezia Giulia (Dente di Cavallo Bianco); Lombardia (Marano Oltrepò, Ottofile Mantovano, Ottofile Pavese, Rostrato di Valchiavenna, and Scagliolo Ticino); Toscana (Rosso Amiata); Trentino‐Alto Adige (Paesan Vigo di Ton); Valle d'Aosta (Châtillon, Entrebin); and Veneto (Biancoperla, Marano). Moreover, Emilia‐Romagna and Toscana landraces exhibited higher levels of admixture, often sharing ancestry with Trentino‐Alto Adige and Veneto. This is consistent with the PCA results. At *K* = 9, a distinct “Marano” group, also highlighted in the PCA, was observed, composed of Quarantino Cremonese, Locale Zambana (closely related but just outside the red circle in Figure 2), Marano Gardolo, Nostrano di Storo, Châtillon, and Marano. At *K* = 13, the degree of admixture decreased, and more landraces were attributed to a single ancestral population (e.g., Dente di Cavallo Bianco, Nano Precoce, and most varieties from Lombardia), while Toscana and Trentino‐Alto Adige still displayed higher heterogeneity.

Overall, the different approaches (admixture, PCA, and clustering analysis) yielded largely consistent results, highlighting both clear groupings and patterns of admixture among the studied landraces.

At *K* = 9, *K* = 11, and *K* = 12 in the admixture analysis (Figures S6 and S7A–C), Rosso Amiata was predominantly assigned to a single ancestral component, with the exception of one admixed individual. This admixed individual was easily identified in the PCA (Figure 2), where it was clearly separated from the other four tightly clustered individuals and associated with the Rostrato‐type cluster. Several other landraces are also noteworthy, including Rustico 1, Rustico 4, Ottofile Garfagnana, Orecchiella, Ottofile Pavese, Giallo Comune, and Nano Precoce. At *K* = 12 (Figure S7C, green bar) and in the phylogenetic dendrogram (Figure S4), these landraces shared a common genetic background and were derived from the same branch. In contrast, the PCA placed them within a broader cluster of individuals, where their differentiation was less apparent. This is likely due to the moderate percentage of variability explained by the principal components.

At *K* = 11 (Figure S7B), landraces from Emilia‐Romagna appeared to be more similar to those from Trentino‐Alto Adige and less so to those from Toscana, yet they still shared genetic material with both regions. This pattern was consistent with the PCA and the dendrogram. At *K* = 9 and *K* = 12, Cinquantino Bianco was assigned to a single ancestral population, shared primarily with landraces from Toscana (with the exception of Rosso Amiata and Rustico 4) and with Trentino‐Alto Adige—particularly Nostrano Vigo di Ton and Paesan Vigo di Ton. This grouping was also evident in the dendrogram, where the three landraces clustered on the same branch, and was partially reflected in the PCA: the five Cinquantino Bianco individuals clustered very closely in PC1 versus PC2, just below Paesan Vigo di Ton, while Nostrano Vigo di Ton was slightly more distant, near the Marano group. In Figure S5A,B, Cinquantino Bianco and Paesan Vigo di Ton consistently clustered together, with Nostrano Vigo di Ton nearby, supporting their close genetic relationship.

An additional group consistently identified across the analyses included the Rostrati (Dente di Cavallo Bianco, Rostrato di Mortara, Rostrato della Valchiavenna), Biancoperla, and Scagliolo Ticino, with the latter appearing the most distinct in both the PCA and admixture analysis.

Genome‐wide, the F_ST_ fixation index was low, with several genomic positions exhibiting high allele fixation, particularly on chromosomes 1, 2, 5, 8, and 10 (Figure S8). The F_ST_ mean across the genome was equal to 0.28, with five out of 2880 SNPs showing values higher than 0.80. The inbreeding coefficient F of the germplasm was highly variable both within and across landraces (Figure S9).

### 3.2 Drivers of Adaptation Across the Climatic Landscape

We assessed the collinearity among bioclimatic variables. Isothermality (BIO3), Temperature Seasonality (BIO4), Minimum Temperature of the Coldest Month (BIO6), Mean Temperature of the Wettest Quarter (BIO8), Mean Temperature of the Driest Quarter (BIO9), Precipitation of the Wettest Month (BIO13), Precipitation Seasonality (BIO15), and Precipitation of the Warmest Quarter (BIO18) were retained for further analyses.

The environmental heterogeneity of the landraces was investigated using PCA (Figure S10). The first three components explain 72.91% of the variability (40.42%, 16.92%, and 15.57%, respectively). Overall, landraces were well distributed along the BIO variables. There was a clear landrace distinction with all landraces from Toscana, the Emilia‐Romagna landrace, and two landraces from Lombardia (Ottofile Pavese and Marano Oltrepò) (left side of Figure S10A).

Variance partitioning based on pRDA demonstrated that climate, geography, and genetic structure collectively accounted for 30% of the total genetic variance across the 140 maize samples (Table 1). Climate alone explained 45% of the partitioned variation, genetic structure contributed 31%, and geographic coordinates explained 11%. In addition, 13% of the explainable variation could not be directly attributed to climate, geography, or genetic structure, indicating some overlap and interaction among these variables.

**TABLE 1:** Effect of climate, geography, and genetic structure on the observed genetic variation among the 140 samples deriving from the 28 different maize Italian landraces decomposed using partial redundancy analysis.

pRDA	Inertia (variance)	*r* ^2^	Adj *r* ^2^	*p* (> *F*)	Proportion of explainable variance	Proportion of total variance
Full model: G ~ clim. + geog. + struct.	242.07	0.30	0.23	0.001***	1.00	0.30
Pure climate: G ~ clim.|(geog. + struct.)	109.38	0.14	0.10	0.001***	0.45	0.14
Pure structure: G ~ struct.|(geog. + clim.)	74.92	0.09	0.08	0.001***	0.31	0.09
Pure geography: G ~ geog.|(struct. + clim.)	26.73	0.03	0.02	0.001***	0.11	0.03
Confounded climate/structure/geography	31.03				0.13	0.04
Total unexplained	555.45					0.70

*Note:* *** means significance at *p* ≤ 0.001.

**FIGURE 3:**
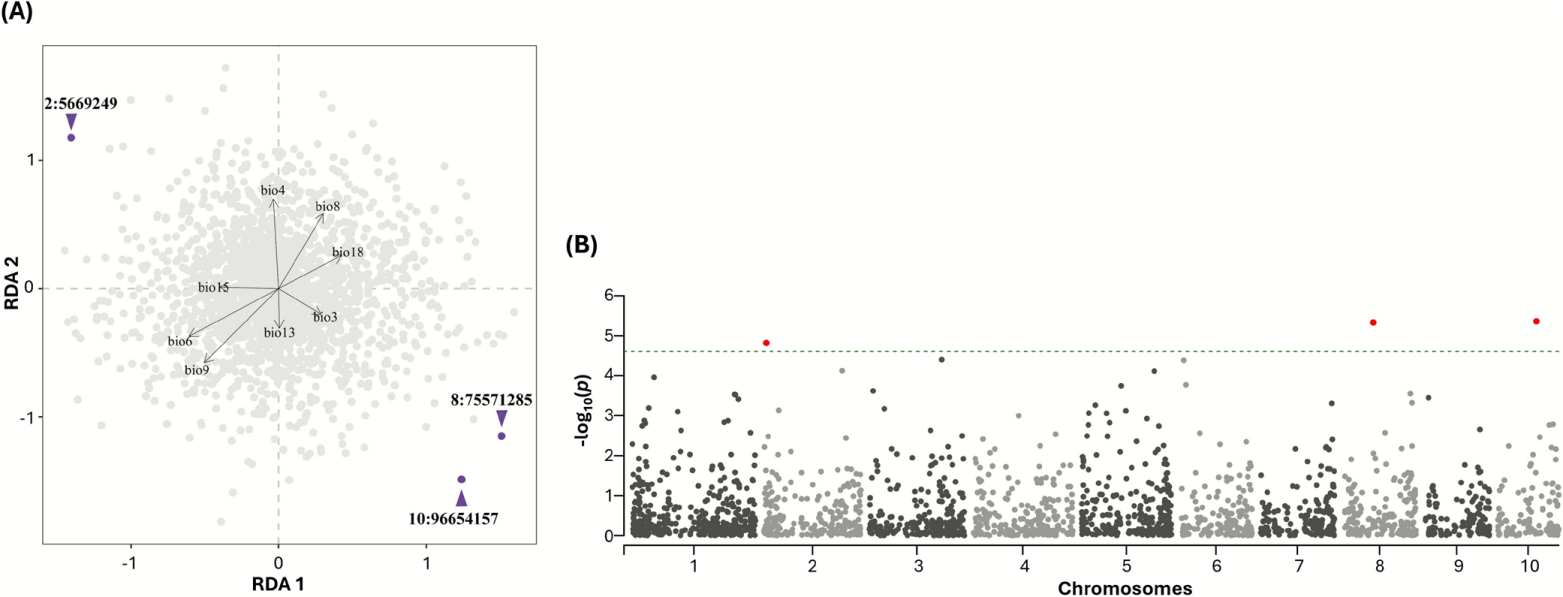
Genotype‐environment association using partial redundancy analysis. (A) Bioclimatic diversity and genotype of 140 samples: Each dot represents a SNP marker; outliers are in violet. (B) Physical position of SNPs on maize genome (*x*‐axis) and level of the statistical association (*y*‐axis) are indicated. Horizontal dashed line indicates Bonferroni threshold, and significant SNPs are in red.

The noncollinear bioclimatic variables were used to search for genotype‐environment associations. We identified three outlier SNPs (Figure 3). Specifically, the SNP 2:5669249 (Figure 3A, RDA1–1.40, RDA2 1.17), located within the gene *Zm00001eb068470*, was mainly driven by Temperature Seasonality (BIO4) and Precipitation Seasonality (BIO15). The SNP 10:96654157 (Figure 3A, RDA1 1.24, RDA2–1.49) within *Zm00001eb418760* was mainly driven by Isothermality (BIO3) and Precipitation of the Wettest Month (BIO13). The SNP 8:75571285 (Figure 3A, RDA1 1.51, RDA2–1.18) appeared to be mainly driven by BIO3 within a non‐coding region.

Genes within the window defined through LD decay were retained and are examined in the Discussion (Table 2).

**TABLE 2:** List of genes within the Linkage disequilibrium window for the significant SNPs deriving from partial redundancy analysis.

SNP ID	±LD‐windows (Kb)	Gene	Function
2:5669249	67.46	*Zm00001eb068480*	Uncharacterized
		*Zm00001eb068500*	Uncharacterized
		*Zm00001eb432150*	Uncharacterized
		*Zm00001eb068520*	AP2‐EREBP‐transcription factor 197
		*Zm00001eb068460*	DNA topoisomerase 2 TOP2A
8:75571285	65.65	*Zm00001eb344580*	Uncharacterized
		*Zm00001eb344590*	Uncharacterized
		*Zm00001eb344600*	ZF‐HD‐transcription factor 11
		*Zm00001eb344610*	Serine/threonine protein kinase 3
10:96654157	77.22	*Zm00001eb418770*	Small subunit ribosomal protein
		*Zm00001eb418780*	Nucleobase‐ascorbate transporter 12
		*Zm00001eb418750*	UNC93‐like protein 1‐ related

### 3.3 Modelling Genetic Diversity and Climate With a Gradient Forest Approach

The genetic diversity and climatic landscape were modelled using a GF approach based on historical climate data and MEMs as predictors, and the LD‐pruned SNPs as response variables. The changes in allelic turnover within the studied materials were best predicted by MEMs, which reflect the geographical origin of the materials. Among these, MEM6 emerged as the most important predictor while BIO15, which represents precipitation seasonality, was identified as the best climatic predictor (Figure S11A). GF analysis was subsequently employed to predict the allelic composition of the climate adaptive loci over the Italian landscape (Figure S11B,C).

**FIGURE 4:**
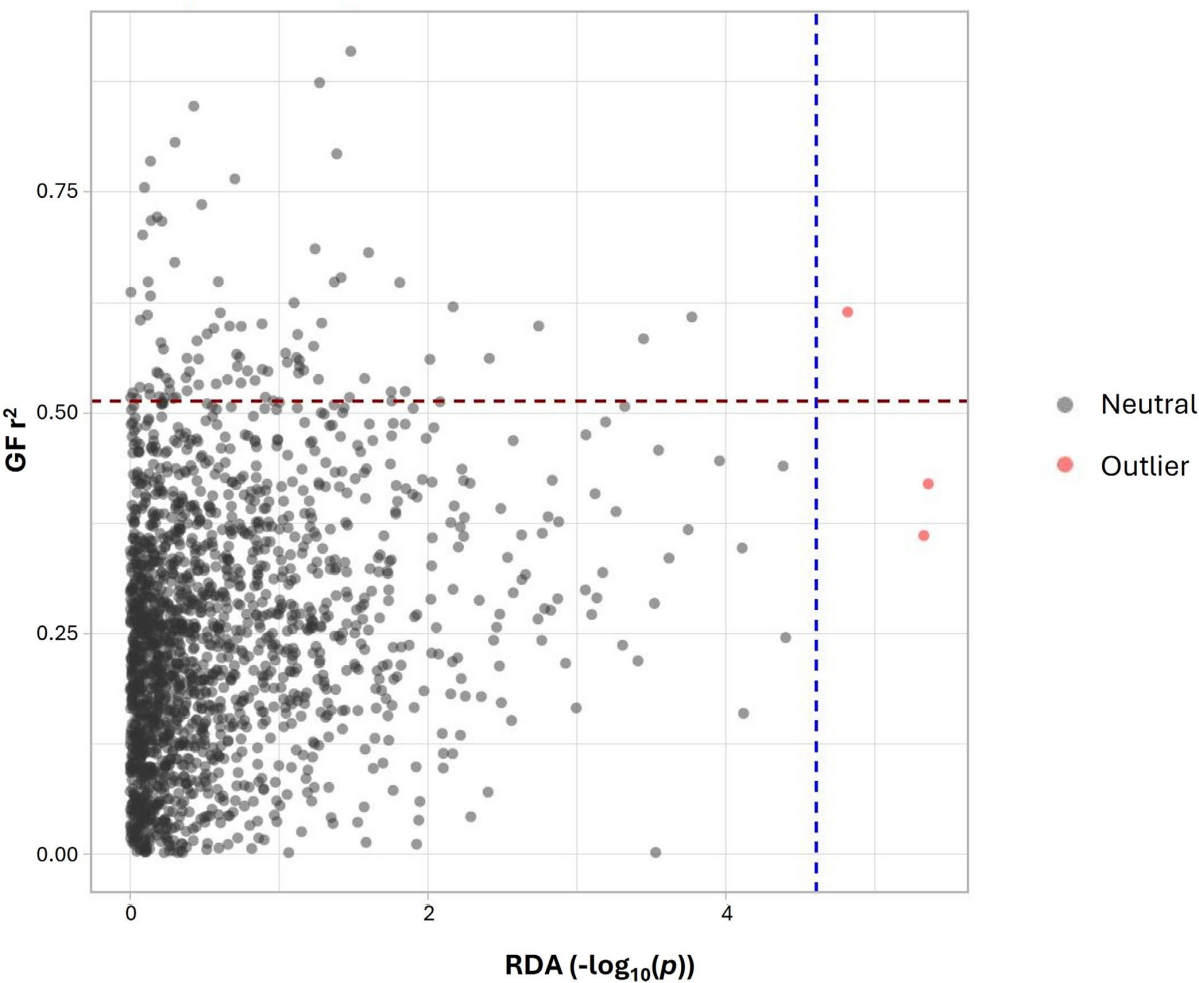
SNP *p*‐value identified with partial redundancy analysis (−log_10_(*p*)) against the weighted *r* ^2^ value of each SNP provided by the gradient forest modelling. The red dots correspond to the SNPs with a significant genotype‐environment association.

The GF analysis yielded *r* ^2^ values for each SNP, representing the proportion of allele frequency variation explained by the climate and geographical variables in the model. A comparison between the *p*‐values of individual SNPs identified using pRDA and the *r* ^2^ values from the GF analysis revealed an SNP (2:5669249) that was significant in the pRDA and among the best‐predicted SNPs by the GF (Figure 4). This SNP was found to be in linkage with a gene encoding an AP2‐EREBP transcription factor, highlighting the potential importance of this genomic region for adaptation.

## 4 Discussion

Maize is one of the leading crops globally owing to its versatility as a human food source and livestock feed, and its potential for various industrial applications (Mereu et al. 2021; Erenstein et al. 2022; Balconi et al. 2024). However, it remains highly susceptible to environmental changes, which increasingly affect modern agriculture. To ensure food security, there is an urgent need for the development of resilient varieties. This can be achieved by exploring the genetic pool of maize landraces, where breeders may identify alleles associated with environmental adaptation. In the present study, an in‐depth analysis of 28 representative Italian maize landraces was conducted using landscape genomic approaches. The research findings can be categorized into two main areas.

The first category encompasses population analysis, providing a clear elucidation of the relationships existing within the investigated germplasm. Specifically, the dendrogram reveals that the most distinctive landrace was Ottofile Mantovano, an eight‐row flint‐type variety. This landrace diverged early from the others and is characterized by a cylindrical ear with fan‐shaped kernels, a distinct plant architecture, long leaves extending up to the last nodes, and a curved growth habit. A group composed of Dente di Cavallo Bianco, Scagliolo del Ticino, Rostrato di Mortara, Rostrato della Valchiavenna, and Biancoperla was also identified. This clustering was partially consistent with the PCA, while admixture analysis indicated Scagliolo del Ticino and Biancoperla to be ancestral to the other landraces. The origin of Rostrato maize (Dente di Cavallo Bianco, Scagliolo del Ticino, Rostrato di Mortara, Rostrato della Valchiavenna) remains poorly understood, although these varieties persist in mountainous areas, where their distinct grain morphology promotes rapid dehydration (Ardenghi et al. 2018; Stagnati et al. 2021; Sangiorgio et al. 2021). Interestingly, within the clustering analysis, the local variety Entrebin remained highly separated. This is supported by the variation highlighted by admixture analysis and is consistent with the history of this variety, which is typically cultivated under strong isolation conditions, likely leading to a high degree of differentiation (Lezzi et al. 2023). The PCA and clustering analysis also showed a cluster of landraces with names or morphology related to the Marano‐type. The admixture analysis better elucidated this outcome. In particular, four landraces were identified as ancestral within the “Marano” group: Marano, Marano Oltrepò, Nostrano di Storo, and Châtillon, with derivatives as Marano Gardolo, Quarantino Cremonese, and Locale Zambana. The term Marano is fairly generic, deriving from the variety improved by Fioretti in 1890 and subsequently named as Marano Vicentino. This variety is an excellent flint maize for polenta production, with tightly packed kernels, medium‐sized to large ears, and an excellent yield. These traits mark the success of the variety in the entire peninsula (Bertolini et al. 2002). Seed self‐production, outcrossing, local adaptation, and renaming may have led to the development of other landraces with similar traits. Nostrano di Storo, wrongly called Marano in the past (Bertolini et al. 2005), is the ancestral population of Locale Zambana and Marano Gardolo, also contributing to other landraces. Clustering analysis separated Marano Oltrepò and Châtillon, the latter characterized by dark‐red kernels, which resulted distinct also by admixture analysis. The apparent “Marano” cluster observed in the PCA likely reflects the limited resolution of the analysis rather than true genetic relatedness among these materials. The PCA determined an additional cluster containing local varieties from Emilia‐Romagna, Toscana, Trentino‐Alto Adige, and Lombardia. Landraces grown in the hills and mountains of the Apennines from Lombardia to Toscana shared a common ancestry, while Cinquantino Bianco from Emilia‐Romagna contributed to landraces from Trentino‐Alto Adige and Toscana. These findings suggest that in the past, the genetic exchange of landraces grown at considerable distances was higher than previously assumed. Farmers commonly brought their own seeds when relocating or marrying, further facilitating this exchange (Troyer 2000; Stagnati et al. 2021). Moreover, a phenotypic selection based on only a few morphological traits was often performed by farmers, producing materials with morphological distinctiveness but genetic similarity (Arteaga et al. 2016).

This study evaluated the drivers of landrace adaptability through pRDA analysis, indicating the relevance of bioclimatic variables in shaping landrace diversity rather than the pure genetic structure. This is consistent with the genetic characterization, which revealed limited differentiation among landraces and a high degree of admixture. Moreover, pRDA analysis identified bioclimatic variables associated with water availability and temperature, leading to the identification of three significant loci. Temperature and rainfall were the two most important drivers shaping landrace selection over time. A common feature of the landrace collection investigated in this study is the conical ear shape. This trait is considered to have a positive adaptive value in areas where water is a limiting factor. In addition, landraces with a strongly conical ear typically reach maturity early in the season, thus adapting well to mountains where the growing season is limited. In contrast, in the Po Valley, where most of the maize was and is actually grown, the possibility to provide irrigation and the higher temperature regime led to the selection of a diversified set of landraces with short to long cycles that were used for both animal and human consumption (Brandolini and Brandolini 2009). The pRDA suggests that germplasm adaptability was driven by a complex interaction of parameters associated with water availability and temperature at the geographical origin of the accessions. The same analysis allowed for the testing and identification of the presence of three significant outlier loci (SNPs) and two positional candidate genes. The first significant SNP, 2:5669249, fell within the gene body of *Zm00001eb068470* that encodes for an uncharacterized protein, and five other genes were within the LD window. The upstream gene *Zm00001eb068520* encodes the AP2‐EREBP‐transcription factor 197 (Harper et al. 2016; Wang et al. 2023). AP2 belongs to the APETALA2/Ethylene Response Factor (AP2/ERF) superfamily, a group of transcription factors that emerged as key regulators of various stress responses (Qi et al. 2023). In maize, drought stress significantly impacts growth, and at least five AP2/ERFs are involved in drought response (Qi et al. 2023). Furthermore, maize AP2/ERFs are involved in salt, waterlogging, and cold stress responses (Qi et al. 2023)—as well as in various biological processes such as growth and development—through mechanisms including transcriptional and post‐translational control (Xie et al. 2019). Chuck et al. (2002) demonstrated that *Branched silkless1* (*bd1*), a member of the AP2/ERF family, is expressed in the ear and tassel tissues of maize. Mutants overexpressing this transcription factor exhibited indeterminate spikelets and produced numerous lateral spikelets, suggesting that AP2/ERFs play a significant role in regulating root, inflorescence, and grain development. Downstream in the LD region, *Zm00001eb068460* encodes for DNA topoisomerase 2 (TOP2A) (Harper et al. 2016). DNA topoisomerases are essential nuclear enzymes involved in DNA metabolism (Bakshi et al. 2001). John et al. (2016) overexpressed DNA topoisomerase 2 in tobacco plants and demonstrated that the overexpression was associated with an enhanced salt stress tolerance and proline and glycine betaine levels, as well as reduced lipid peroxidation, while topoisomerase IV was linked to reactive oxygen species response in *Arabidopsis* (Šimková et al. 2012). Although SNP 8:75571285 fell within a non‐genic region, several interesting genes were observed in the LD windows upstream of the SNP position. Specifically, *Zm00001eb344600* encodes a zinc‐finger homeodomain transcription factor 11 (zhd11) (Harper et al. 2016). Maize ZHDs play an important role in the activation of stress‐mediated pathways by stresses such as drought, high salinity levels, and high temperatures (Jia et al. 2006; Khatun et al. 2017; Shalmani et al. 2019). They are also key in the growth and development of floral and vegetative tissues (Kozaki et al. 2004; Islam et al. 2022). A second gene, *Zm00001eb344610*, encodes for serine/threonine protein kinase 3. In maize, the function of such enzymes that use ATP to phosphorylate other proteins within the cell is poorly characterized (Azad and Alemzadeh 2017). Studies suggest their implication in the control of floret number and ear length (Jia et al. 2020), pollen grain formation, and maturation (Huang et al. 2017), as well as their association with the cell signal transduction of maize in response to drought stress (Li et al. 2009).

Finally, although gene *Zm00001eb418760* (SNP 10:96654157) encodes for an uncharacterized protein, three additional genes were located within the LD window. Specifically, *Zm00001eb418770* encodes a small subunit ribosomal protein; *Zm00001eb418780* a nucleobase‐ascorbate transporter 12 in *Z. mays* , and *Zm00001eb418750* an UNC93‐like protein 1‐related protein (Harper et al. 2016). While this specific protein has not yet been characterized, *UNC93‐like* genes have been found to regulate asexual reproduction in *Arabidopsis*, with UNC93‐like transcripts present only in seed and silique tissues. This suggests their crucial role in reproductive tissue development. Furthermore, *UNC93‐like* genes may have unknown roles in fruit development (Cordle et al. 2012; Schaeffer et al. 2016).

Two common and recurring characteristics among the genes within the LD window of the aforementioned three significant SNPs are their involvement in the growth and development of the reproductive organs of the plant and their role in response to drought stress. Considering that the ultimate goal of plant breeding is to achieve high performance, particularly in terms of yield, even under adverse conditions (Hallauer 2007), the identification of genes directly involved in yield‐related processes is particularly promising.

The results of the GF analysis revealed predominant drivers of landrace adaptation. The adaptive environments within the right side of the Po Valley are driven by BIO3 (Isothermality) and BIO15 (Precipitation Seasonality), while those on the left side of the Po Valley and the eastern side of Emilia‐Romagna are driven by BIO8 (Mean Temperature of Wettest Quarter). The Apennines chain and Toscana appear to be primarily influenced by BIO6 (Minimum Temperature of Coldest Month) and BIO9 (Mean Temperature of Driest Quarter). Adaptive environments in the other mountain areas, such as Valle d'Aosta and the Alps, appear to be driven by BIO13 (Precipitation of Wettest Month) and BIO4 (Temperature Seasonality). The patterns identified by GF analysis reflect the climatic trends: maize landraces grown in the right side of the Po Valley are more exposed to hot and dry periods compared to those grown on the left side, while those in the Apennines are characterized by short seasons and drought susceptibility. Finally, the Alps are generally characterized by a short growing season and limited water availability (Brandolini and Brandolini 2009; Stagnati et al. 2022).

A key finding of the GF analysis is the confirmation of the pRDA results. The SNP 2:5669249, which was identified as significant by pRDA and found in linkage with the gene coding for APETALA2, was among the best‐predicted SNPs by GF. This reinforces the importance of local resources in future breeding programs.

## 5 Conclusion

This study characterized 140 individuals from 28 maize landraces collected across the most representative locations of Northern‐Central Italy to identify genetic markers associated with environmental adaptation. The landscape genomics approach allowed for the identification of three SNPs significantly associated with bioclimatic variables. Further analyses of the genes within the LD windows surrounding these markers detected a gene encoding the AP2‐EREBP transcription factor, which is known to regulate key pathways involved in plant development and stress responses, potentially impacting yield. This gene plays a potentially critical role in maize adaptation to environmental stresses such as flooding and heat. Our results lay the foundation for breeding programs focused on enhancing local adaptation and leveraging landraces to improve stress tolerance, thereby ensuring stable yields in the face of climate change.

## Funding

This work is part of the project NODES, which has received funding from the MUR–M4C2 1.5 of PNRR with grant agreement no. (ECS00000036).

## Conflicts of Interest

The authors declare no conflicts of interest.

## Supporting information


**Table S1:** Detailed information regarding individual landrace names, precise collection points, and geographic coordinates of the original collection sites of the 28 Italian landraces under study. Germplasm collection was retrieved from Institut Agricole Régional (IAR); Council for Agricultural Research and Economics, Research Centre for Cereal and Industrial Crops (CREA‐CI); Agenzia Regionale per lo Sviluppo e l'Innovazione del Settore Agricolo e Forestale (A.R.S.I.A.); Department of Earth and Environmental Sciences of Università degli Studi di Pavia (UNIPV); Department of Sustainable Crop Production of Università Cattolica del Sacro Cuore (UCSC). Whenever available, references reporting morphological description and additional information were added.
**Table S2:** List of the bioclimatic variables and their respective codes derived from WorldClim (Fick and Hijmans 2017).
**Table S3:** Linkage disequilibrium half‐decay.
**Figure S1:** SNP density plot across the 10 chromosomes of maize representing number of SNPs within 1 Mb window size. The horizontal axis represents the chromosome length in Mb. Different colors correspond to SNP density.
**Figure S2:** Genome‐wide linkage disequilibrium (LD) estimated from 140 maize individuals deriving from the 28 different maize Italian landraces. LD values plotted in a rolling window along each chromosome, with line colors corresponding to the legend. Black arrowheads mark centromere positions (A). LD decay as a function of physical distance between markers, expressed in megabases (Mb) (B). The difference in absolute LD values between the two panels reflects the use of distinct estimation methods: raw rolling‐window calculations in panel A and interpolated values in panel B.
**Figure S3:** Kinship matrix represented as heatmap of pairwise similarities among the 140 individuals deriving from the 28 different maize Italian landraces. Red and blue colors represent low and high similarities, respectively. On the left and top side, a hierarchical tree represents these relationships. Samples were ordered according to R base *hclust()* function.
**Figure S4:** Dendrogram of the 140 individuals deriving from the 28 different maize Italian landraces. Colors highlight landraces' region of origin.
**Figure S5:** Principal Component Analysis (PCA) score plot based on the LD‐pruned SNP dataset of 140 samples. (A) Component 2 versus Component 3. (B) Component 3 versus Component 1.
**Figure S6:** Plot of ADMIXTURE cross validation error from *K* = 2 through *K* = 30. *K* = 12 was chosen to analyze SNP data, being the value that better minimized the error.
**Figure S7:** Population genetic structure at *K* = 9, 11, 12 and 13 of the 140 individuals from the 28 maize landraces evaluated in the present study. Different colors correspond to different ancestral populations.
**Figure S8:** Fixation index per site (F_ST_) computed for each of the 2880 SNPs derived via LD‐pruning.
**Figure S9:** Mean and standard deviation of the inbreeding coefficient (F) computed for each of the 2880 SNPs derived via LD‐pruning and divided per landrace.
**Figure S10:** Principal component analysis (PCA) of maize landraces based on altitude and eight non‐collinear bioclimatic variables. Plots show pairwise combinations of the first three principal components: (A) PC1 versus PC2, (B) PC2 versus PC3, and (C) PC3 versus PC1. Each point represents a landrace, with colors distinguishing individual landraces and point shapes indicating geographic regions. Red arrows represent the loadings of the bioclimatic variables, pointing in the direction of increasing values and scaled according to their contribution to the components. The landraces Locale Zambana and Scagliolo Locale Zambana (PC1 = 1.253, PC2 = 0.129, PC3 = 0.369) share identical scores and are therefore perfectly overlapping, with only the symbol of Scagliolo Locale Zambana visible. Similarly, Nostrano Vigo di Ton and Paesan Vigo Ton (PC1 = 2.509, PC2 = −0.915, PC3 = −1.081) overlap completely, and only the symbol corresponding to Nostrano Vigo di Ton is displayed.
**Figure S11:** Bioclimatic (BIOs) and Moran's Eigenvector Maps (MEMs) predictors explaining genomic variation in Northern‐Central Italian landraces. (A) Ranked accuracy importance, in terms of predictive power, of the top bioclimatic and spatial variables, based on gradient forest (GF) analysis. (B) Biplot of the biological space represented by principal components of the transformed grid. The first three principal components were transformed into a defined RGB color palette, where red is defined by values of PC1 + PC2, green by negative values of PC2, and blue by PC3 + PC2 − PC1. Different adaptive environments across the cropping area are displayed, where similar colors represent similar alleles at the predicted loci (*r* ^2^ > 0); in the biplot, axes report the portion of bioclimatic variance (%) explained by the PCs of the transformed grid. (C) GF‐transformed bioclimatic variables across the cropping area of maize landrace collection. Colors are based on the biplot of the biological space as in panel (B). Black dots represent landraces' collection sites.

## Data Availability

All data generated or analysed during this study are included in the published article and its supporting information S1. The VCF data associated with this study are available in the European Variation Archive (EVA) under the project code PRJEB101803, and will be accessible for download at the following link: https://www.ebi.ac.uk/eva/?eva‐study=PRJEB101803.
