# Supplementary material for: Leveraging Knowledge of Traditional Italian Maize Landrace Diversity to Identify Signals of Local Adaptation
Source: Evol Appl. 2026 Feb 13;19(2):e70186. doi: 10.1111/eva.70186 (PMC12903548; doi:10.1111/eva.70186)

***Evolutionary Applications* Supporting Information**

**Article title:** LEVERAGING KNOWLEDGE OF TRADITIONAL ITALIAN MAIZE LANDRACE DIVERSITY TO IDENTIFY SIGNALS OF LOCAL ADAPTATION

**Authors:** Alessandra Lezzi^1*^, Lorenzo Stagnati^1,2*^, Leonardo Caproni^3^, Matteo Dell’Acqua^3^, Matteo Busconi^1,2^, Alessandra Lanubile^1,2^, Adriano Marocco^1,2^

1. Department of Sustainable Crop Production, Università Cattolica Del Sacro Cuore, Via Emilia Parmense 84, 29122, Piacenza, Italy
2. Research Centre for Biodiversity and Ancient DNA, Università Cattolica Del Sacro Cuore, Via Emilia Parmense 84, 29122, Piacenza, Italy
3. Center of Plant Sciences, Scuola Superiore Sant'Anna, Pisa, Italy

^*^Alessandra Lezzi and Lorenzo Stagnati are equally contributing authors.

Correspondence: Alessandra Lanubile

Email: alessandra.lanubile@unicatt.it

The following Supporting Information is available for this article:

**Table S1**: Detailed information regarding individual landrace names, precise collection points, and geographic coordinates of the original collection sites of the 28 Italian landraces under study. Germplasm collection was retrieved from Institut Agricole Régional (IAR); Council for Agricultural Research and Economics, Research Centre for Cereal and Industrial Crops (CREA-CI); Agenzia Regionale per lo Sviluppo e l’Innovazione del Settore Agricolo e Forestale (A.R.S.I.A.); Department of Earth and Environmental Sciences of Università degli Studi di Pavia (UNIPV); Department of Sustainable Crop Production of Università Cattolica del Sacro Cuore (UCSC). Whenever available, references reporting morphological description and additional information were added.

| Access ID | Landrace name | Collection site name | Region | Province | Latitude | Longitude | Seed source | Reference |
| --- | --- | --- | --- | --- | --- | --- | --- | --- |
| NVT | Nostrano Val Tidone | Borgonovo Val Tidone | Emilia-Romagna | Piacenza | 45.0145 | 9.4585 | UCSC | / |
| Va221 | Turco | Borgo Val di Taro | Emilia-Romagna | Parma | 44.4907 | 9.7574 | CREA-CI | Brandolini and Brandolini 2006; Di Pasquale et al. 2024 |
| Va225 | Nano Precoce | Ottone | Emilia-Romagna | Piacenza | 44.6238 | 9.3285 | CREA-CI | Brandolini and Brandolini 2006; Di Pasquale et al. 2024 |
| Va216 | Giallo Comune | Santa Sofia | Emilia-Romagna | Forlì | 43.9487 | 11.914 | CREA-CI | Brandolini and Brandolini 2006; Di Pasquale et al. 2024 |
| Va220W | Cinquantino Bianco | Modena | Emilia-Romagna | Modena | 44.6688 | 10.9595 | CREA-CI | Brandolini and Brandolini, 2006; Di Pasquale et al. 2024 |
| R3 | Dente di Cavallo Bianco | Dolegna del Collio | Friuli-Venezia Giulia | Gorizia | 46.0316 | 13.4813 | UNIPV | Ardenghi et al. 2018; Stagnati et al. 2021 |
| QC | Quarantino Cremonese | Regona (Pizzighettone) | Lombardia | Cremona | 45.2058 | 9.8215 | UCSC | Stagnati et al. 2022 |
| R17 | Rostrato Valchiavenna | Prata Camportaccio | Lombardia | Sondrio | 46.309 | 9.389 | UNIPV | Ardenghi et al. 2018; Stagnati et al. 2021 |
| R16 | Rostrato Mortara | Mortara | Lombardia | Pavia | 45.257 | 8.7628 | UNIPV | Ardenghi et al. 2018; Stagnati et al. 2021 |
| A/88-PV-1097 | Ottofile Pavese | Varzi | Lombardia | Pavia | 44.8206 | 9.1883 | UNIPV | Stagnati et al. 2022 |
| MN-976 | Ottofile Mantovano | Motta Baluffi | Lombardia | Cremona | 45.0539 | 10.2512 | UNIPV | Stagnati et al. 2022 |
| R2 | Scagliolo Ticino | Robecchetto Con Induno | Lombardia | Milano | 45.5361 | 8.7726 | UNIPV | Ardenghi et al. 2018 |
| 1915 | Marano Oltrepo' | Santa Maria della Versa | Lombardia | Pavia | 44.9899 | 9.3047 | UNIPV | Stagnati et al. 2022 |
| Va1292 | Rosso Amiata | Castel del Piano | Toscana | Grosseto | 42.8962 | 11.549 | A.R.S.I.A. | / |
| Va1281 | Rustico_1 | Arezzo | Toscana | Arezzo | 43.4499 | 11.8495 | A.R.S.I.A. | / |
| Va1284 | Rustico_4 | Anghiari | Toscana | Arezzo | 43.542 | 12.031 | A.R.S.I.A. | / |
| Va1290 | Orecchiella | Lucca | Toscana | Lucca | 43.8501 | 10.4809 | A.R.S.I.A. | <http://germoplasma.regione.toscana.it> |
| Va1291 | Ottofile Garfagnana | Piezza Gallicano | Toscana | Lucca | 44.0922 | 10.4556 | A.R.S.I.A. | <http://germoplasma.regione.toscana.it> |
| Va148 | Paesan Vigo di Ton | Vigo di Ton | Trentino-Alto Adige | Trento | 46.2688 | 11.0912 | CREA-CI | Bertolini et al. 2005; Brandolini and Brandolini 2006 |
| Va1214 | Nostrano Storo | Storo | Trentino-Alto Adige | Trento | 45.8507 | 10.5688 | CREA-CI | Bertolini et al. 2005; Brandolini and Brandolini 2006 |
| Va153 | Locale Zambana | Zambana | Trentino-Alto Adige | Trento | 46.149 | 11.1075 | CREA-CI | Bertolini et al. 2005; Brandolini and Brandolini 2006 |
| Va154 | Marano Gardolo | Gardolo | Trentino-Alto Adige | Trento | 46.0959 | 11.1063 | CREA-CI | Bertolini et al. 2005; Brandolini and Brandolini 2006 |
| Va145 | Nostrano Vigo di Ton | Vigo di Ton | Trentino-Alto Adige | Trento | 46.2688 | 11.0912 | CREA-CI | Bertolini et al. 2005; Brandolini and Brandolini 2006 |
| Va555 | Scagliolo Locale Zambana | Zambana | Trentino-Alto Adige | Trento | 46.149 | 11.1075 | CREA-CI | Bertolini et al. 2005; Brandolini and Brandolini 2006 |
| VDA3 | Chatillon | Chatillon | Valle d'Aosta | Aosta | 45.7497 | 7.627 | IAR | Lezzi et al. 2024 |
| VDA4 | Entrebin | Entrebin (Aosta) | Valle d'Aosta | Aosta | 45.7574 | 7.3044 | IAR | Lezzi et al. 2024 |
| Marano | Marano | Isola Vicentina | Veneto | Vicenza | 45.629 | 11.4631 | UCSC | Brandolini and Brandolini 2006 |
| Biancoperla | Bianco Perla | Montecchio Precalcino | Veneto | Vicenza | 45.6797 | 11.5724 | UCSC | Brandolini and Brandolini 2006 |

**Table S2**: List of the bioclimatic variables and their respective codes derived from WorldClim (Fick & Hijmans 2017).

| CODE | BIOCLIMATIC VARIABLE |
| --- | --- |
| BIO1 | Annual Mean Temperature |
| BIO2 | Mean Diurnal Range (Mean of monthly (max temp - min temp)) |
| BIO3 | Isothermality (BIO2/BIO7) (×100) |
| BIO4 | Temperature Seasonality (standard deviation ×100) |
| BIO5 | Max Temperature of Warmest Month |
| BIO6 | Min Temperature of Coldest Month |
| BIO7 | Temperature Annual Range (BIO5-BIO6) |
| BIO8 | Mean Temperature of Wettest Quarter |
| BIO9 | Mean Temperature of Driest Quarter |
| BIO10 | Mean Temperature of Warmest Quarter |
| BIO11 | Mean Temperature of Coldest Quarter |
| BIO12 | Annual Precipitation |
| BIO13 | Precipitation of Wettest Month |
| BIO14 | Precipitation of Driest Month |
| BIO15 | Precipitation Seasonality (Coefficient of Variation) |
| BIO16 | Precipitation of Wettest Quarter |
| BIO17 | Precipitation of Driest Quarter |
| BIO18 | Precipitation of Warmest Quarter |
| BIO19 | Precipitation of Coldest Quarter |

**Table S3**: Linkage disequilibrium half-decay.

| Chromosome | max | Mb | Kb |
| --- | --- | --- | --- |
| 1 | 0.459833 | 0.019939 | 19.939 |
| 2 | 0.45985 | 0.067464 | 67.464 |
| 3 | 0.459851 | 0.062003 | 62.003 |
| 4 | 0.459855 | 0.147373 | 147.373 |
| 5 | 0.459822 | 0.018899 | 18.899 |
| 6 | 0.459442 | 0.001237 | 1.237 |
| 7 | 0.459852 | 0.081459 | 81.459 |
| 8 | 0.459851 | 0.06565 | 65.65 |
| 9 | 0.459849 | 0.101485 | 101.485 |
| 10 | 0.459852 | 0.077217 | 77.217 |

**Figure S1**: SNP density plot across the 10 chromosomes of maize representing number of SNPs within 1 Mb window size. The horizontal axis represents the chromosome length in Mb. Different colors correspond to SNP density.


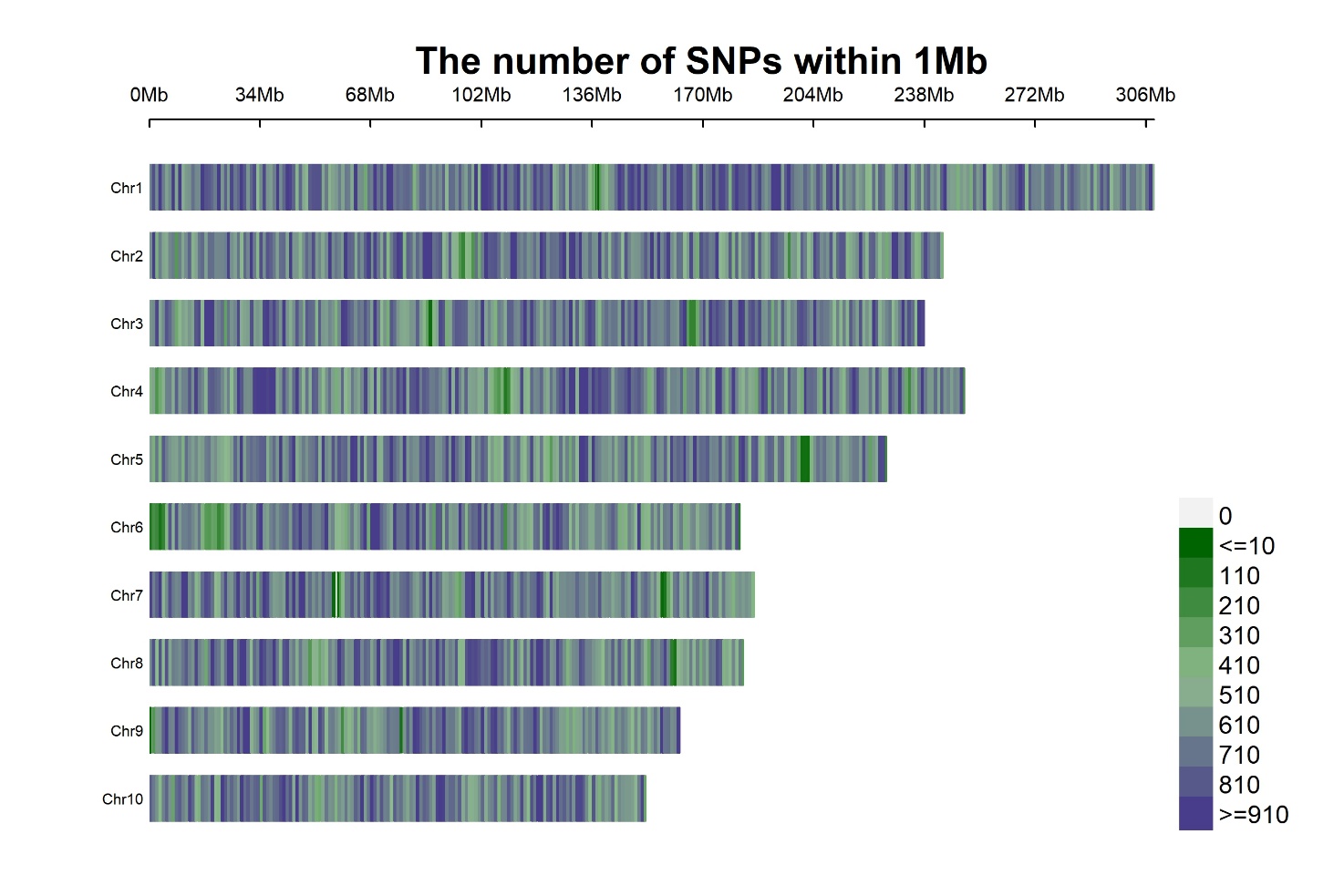


**Figure S2**: Genome-wide linkage disequilibrium (LD) estimated from 140 maize individuals deriving from the 28 different maize Italian landraces. LD values plotted in a rolling window along each chromosome, with line colors corresponding to the legend. Black arrowheads mark centromere positions (A). LD decay as a function of physical distance between markers, expressed in megabases (Mb) (B). The difference in absolute LD values between the two panels reflects the use of distinct estimation methods: raw rolling-window calculations in panel A and interpolated values in panel B.


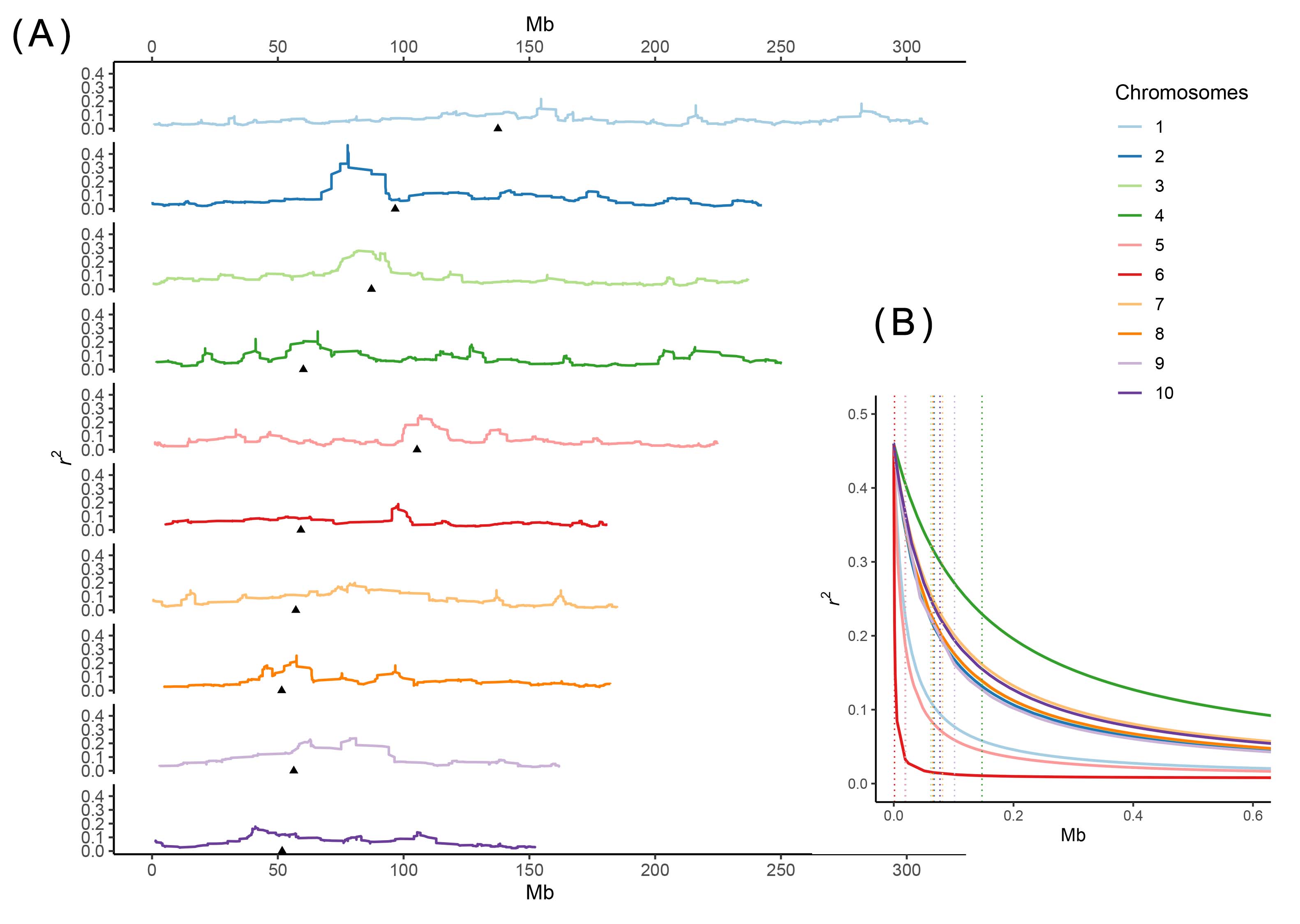


**Figure S3**: Kinship matrix represented as heatmap of pairwise similarities among the 140 individuals deriving from the 28 different maize Italian landraces. Red and blue colors represent low and high similarities, respectively. On the left and top side, a hierarchical tree represents these relationships. Samples were ordered according to R base *hclust()* function.


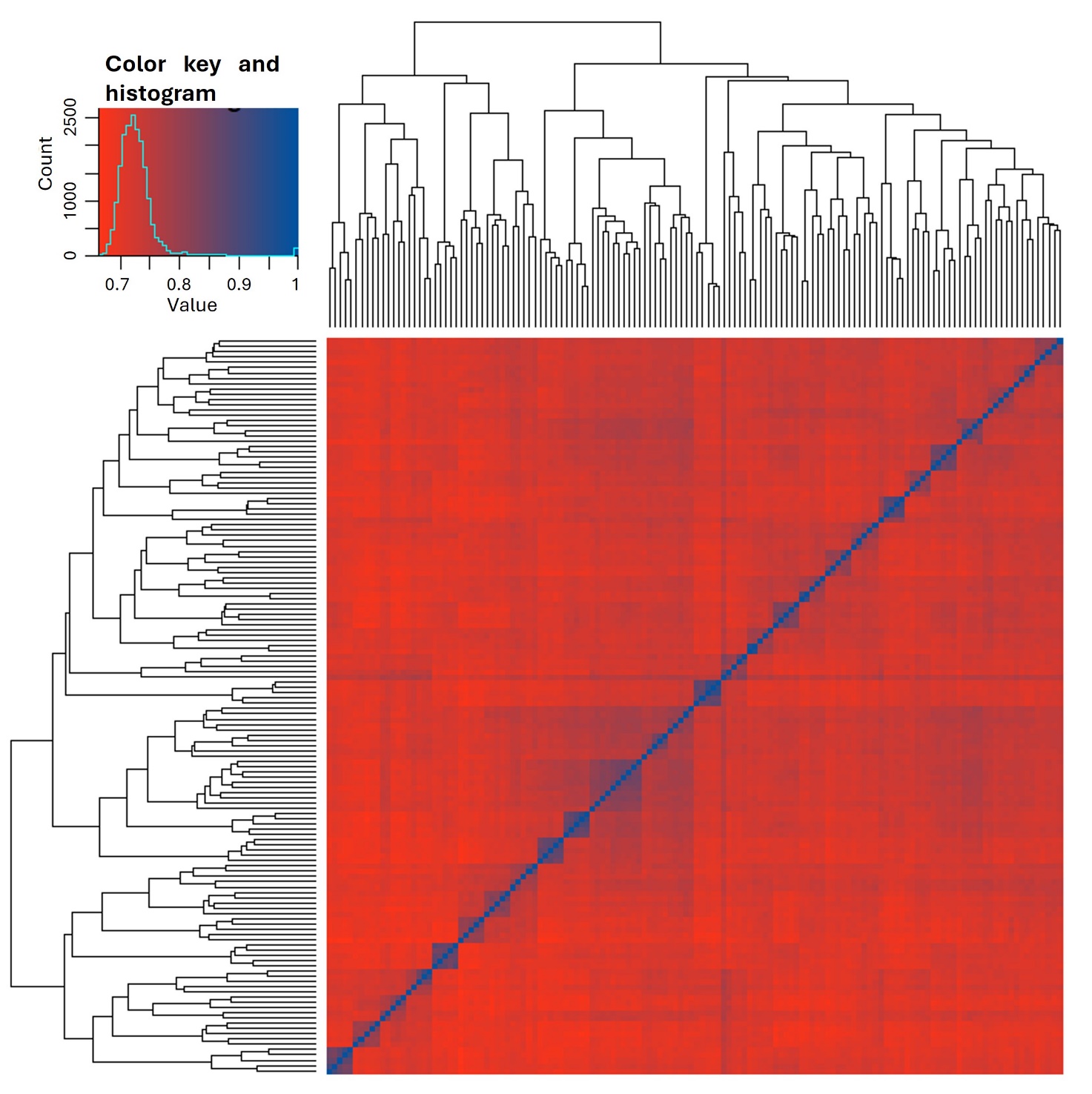


**Figure S4**: Dendrogram of the 140 individuals deriving from the 28 different maize Italian landraces. Colors highlight landraces’ region of origin.

**
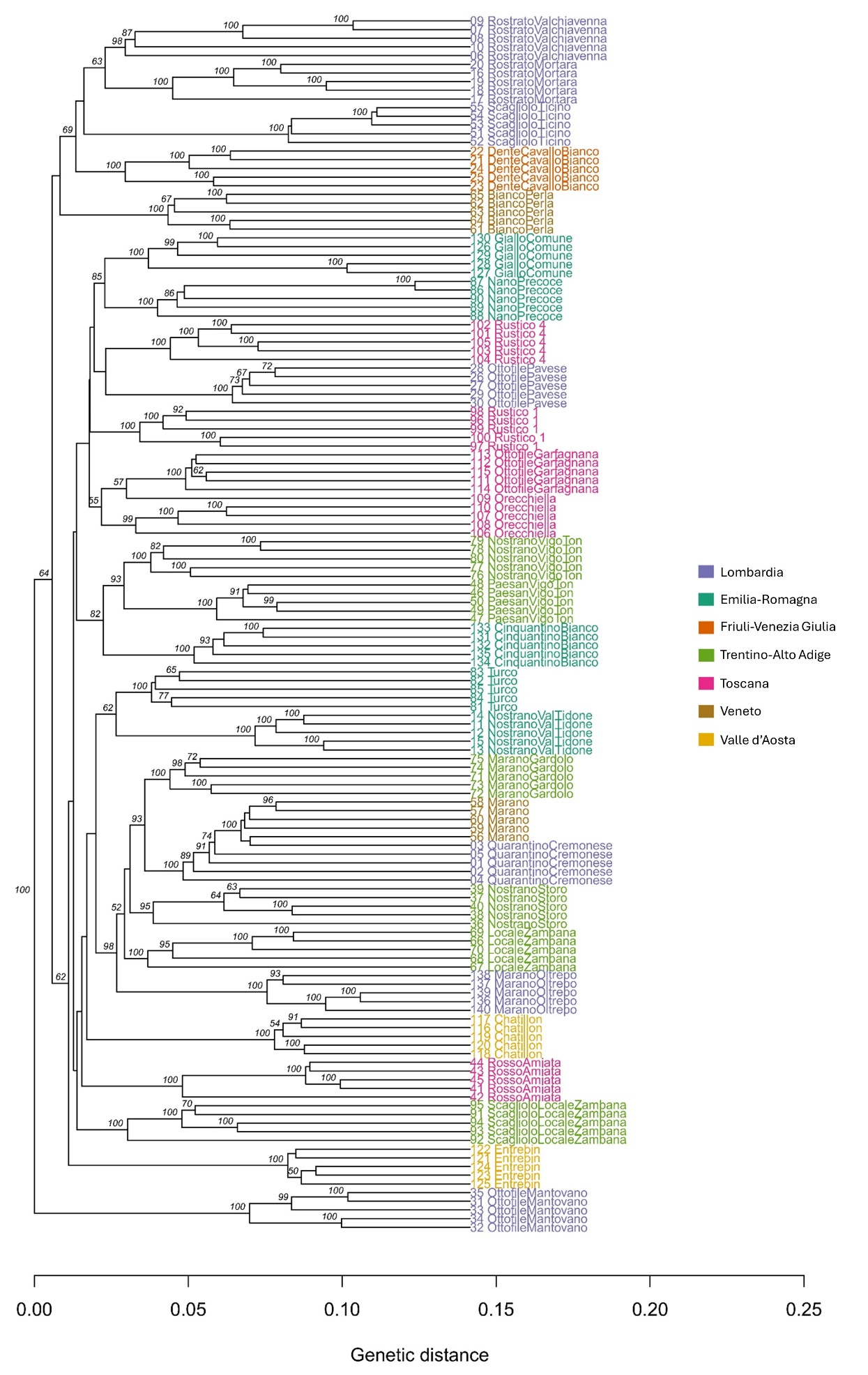
**

**Figure S5**: Principal Component Analysis (PCA) score plot based on the LD-pruned SNP dataset of 140 samples. (A) Component 2 vs. Component 3. (B) Component 3 vs. Component 1.


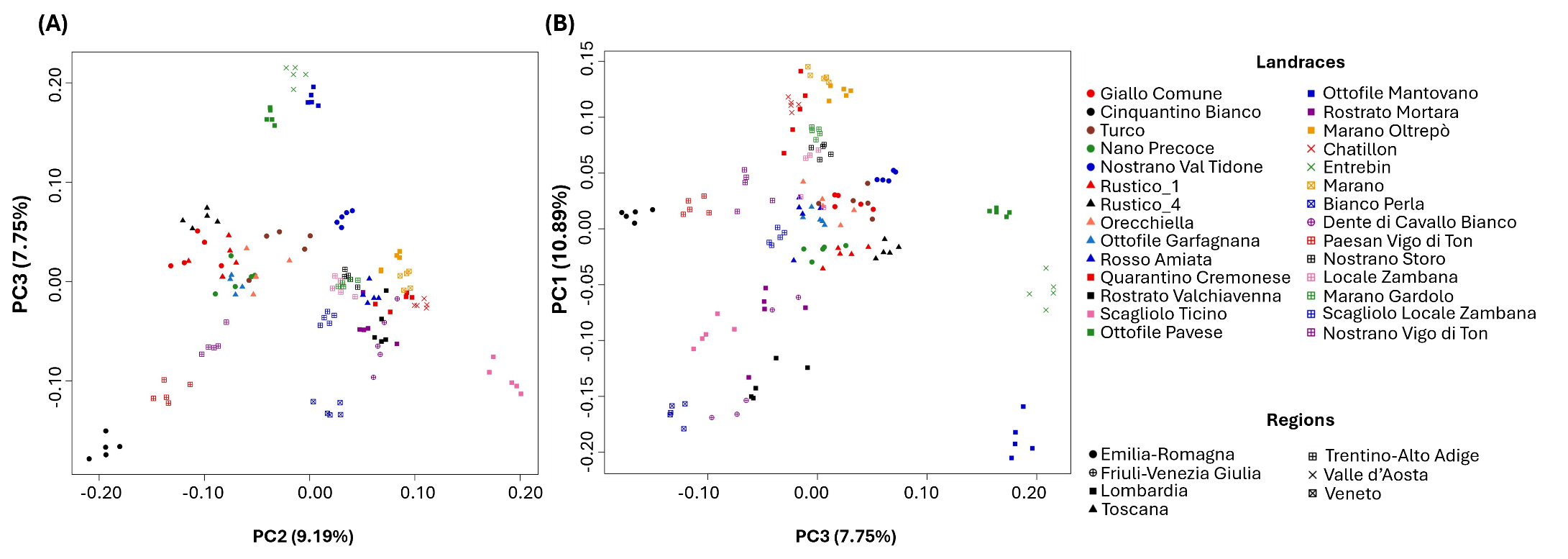


**Figure S6**: Plot of ADMIXTURE cross validation error from K=2 through K=30. K=12 was chosen to analyze SNP data, being the value that better minimized the error.


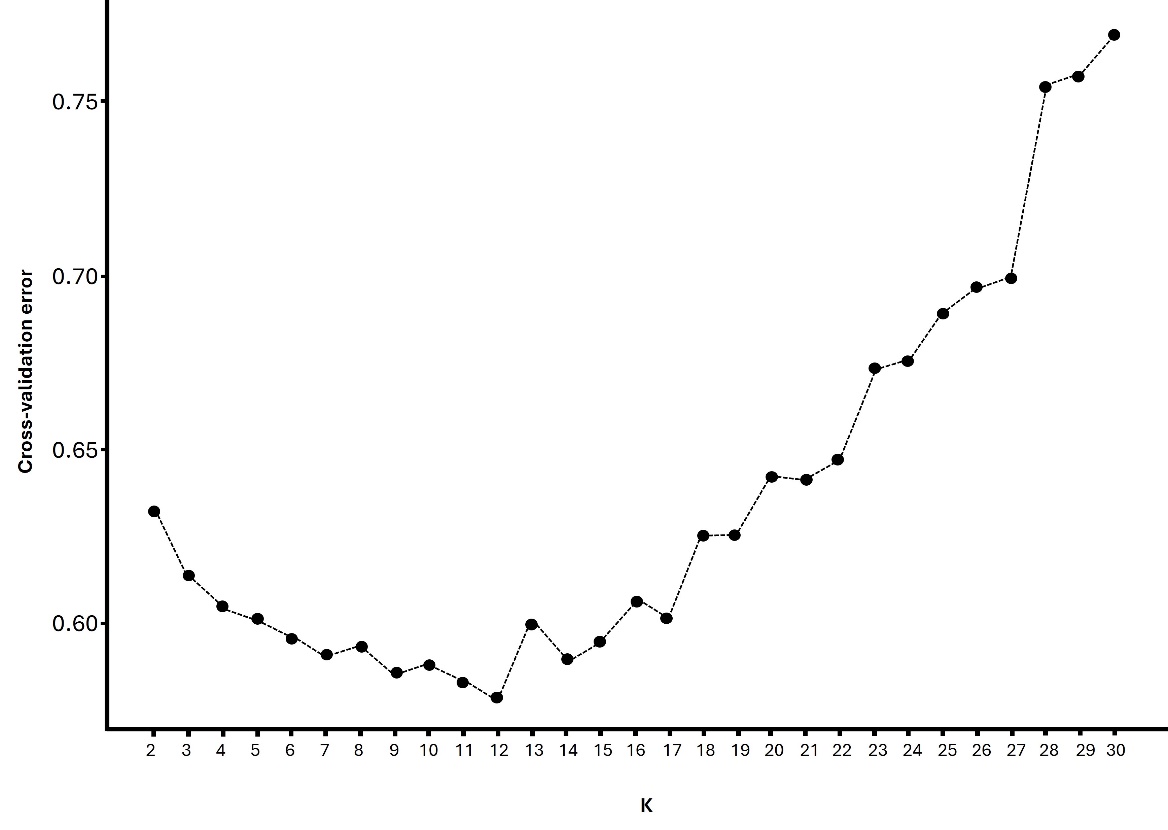


**Figure S7**: Population genetic structure at K = 9, 11, 12 and 13 of the 140 individuals from the 28 maize landraces evaluated in the present study. Different colors correspond to different ancestral populations.


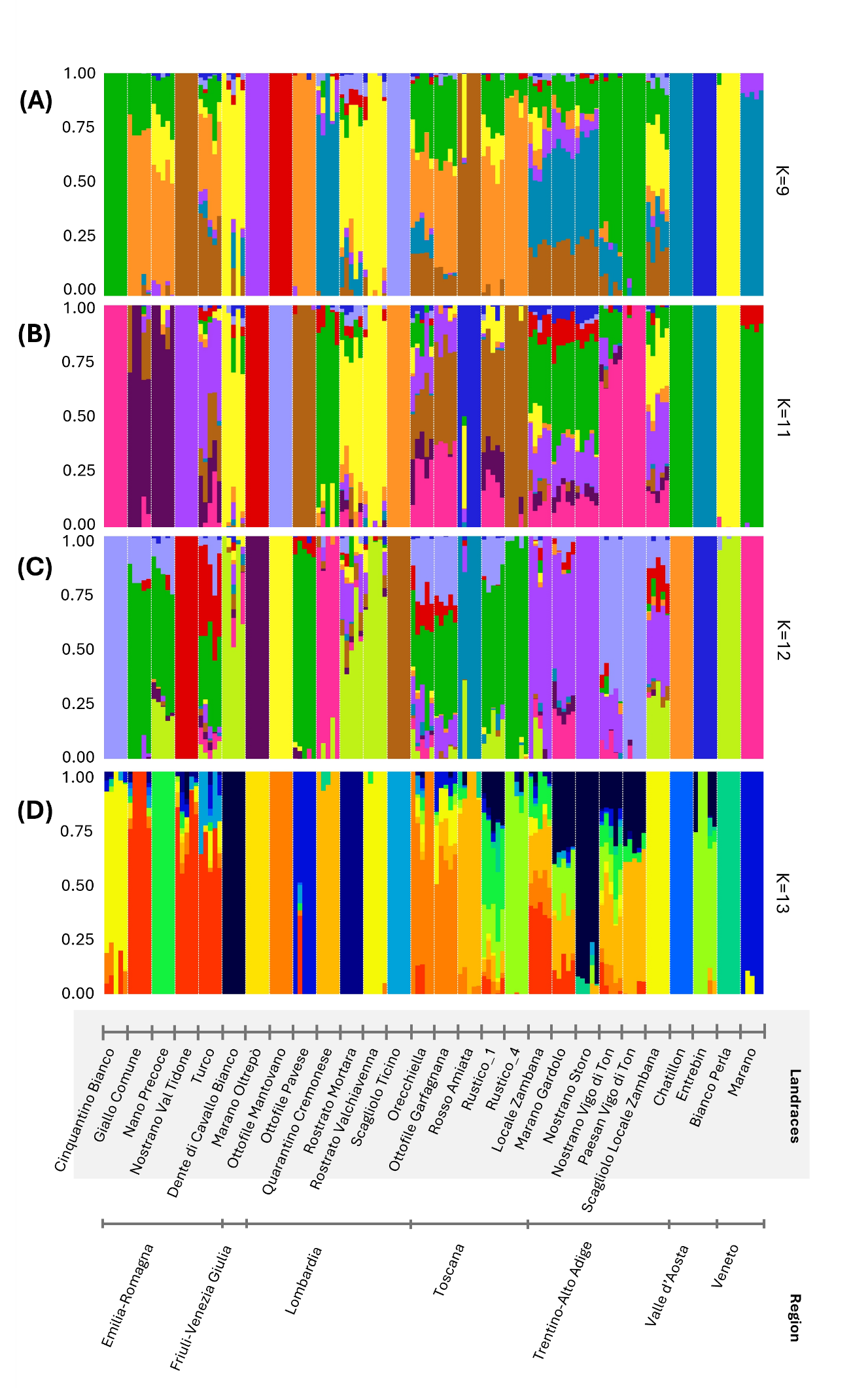


**Figure S8:** Fixation index per site (F_ST_) computed for each of the 2,880 SNPs derived via LD-pruning.


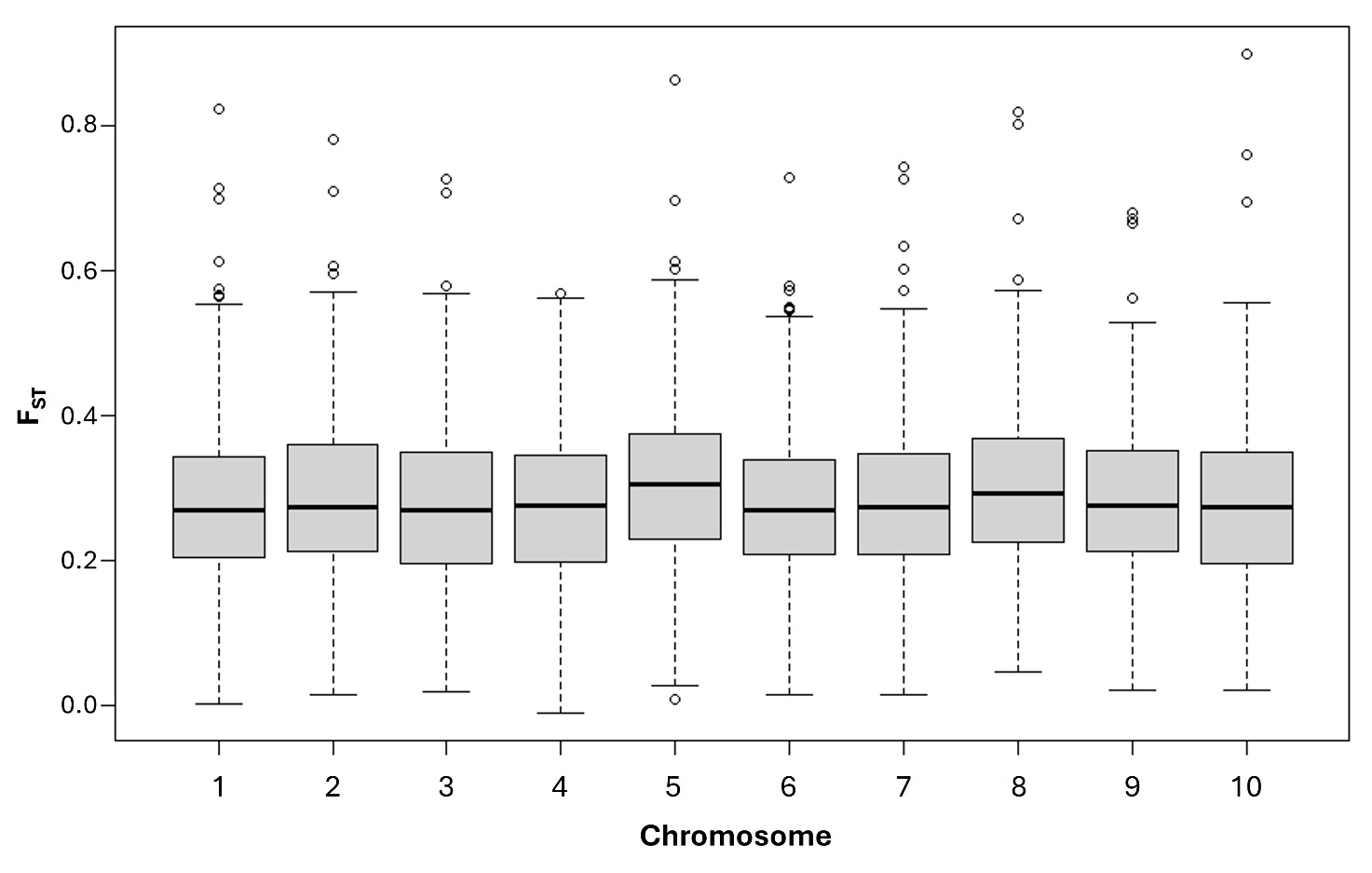


**Figure S9:** Mean and standard deviation of the inbreeding coefficient (F) computed for each of the 2,880 SNPs derived via LD-pruning and divided per landrace.


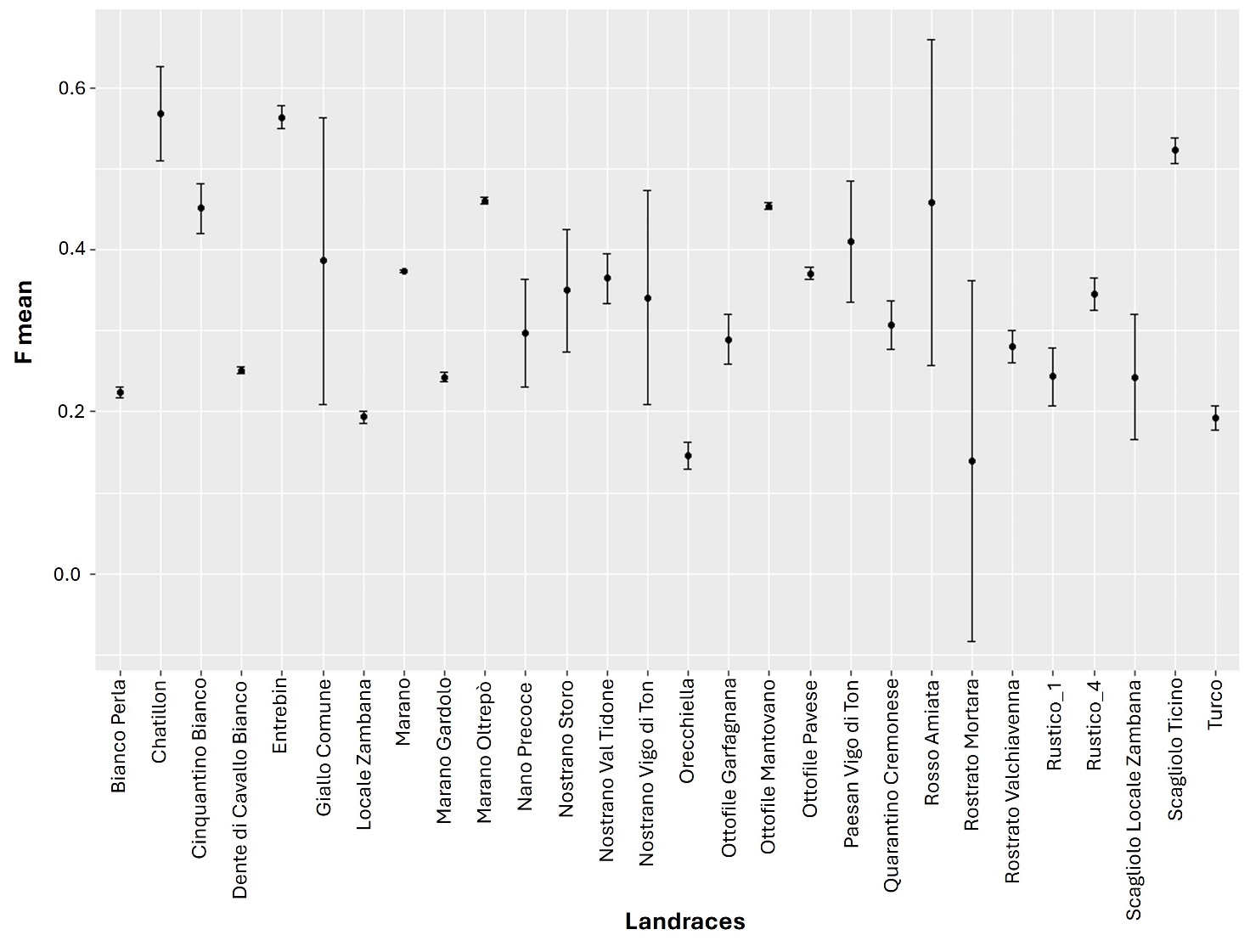


**Figure S10**: Principal component analysis (PCA) of maize landraces based on altitude and eight non-collinear bioclimatic variables. Plots show pairwise combinations of the first three principal components: (A) PC1 vs. PC2, (B) PC2 vs. PC3, and (C) PC3 vs. PC1. Each point represents a landrace, with colors distinguishing individual landraces and point shapes indicating geographic regions. Red arrows represent the loadings of the bioclimatic variables, pointing in the direction of increasing values and scaled according to their contribution to the components. The landraces Locale Zambana and Scagliolo Locale Zambana (PC1 = 1.253, PC2 = 0.129, PC3 = 0.369) share identical scores and are therefore perfectly overlapping, with only the symbol of Scagliolo Locale Zambana visible. Similarly, Nostrano Vigo di Ton and Paesan Vigo Ton (PC1 = 2.509, PC2 = –0.915, PC3 = –1.081) overlap completely, and only the symbol corresponding to Nostrano Vigo di Ton is displayed.


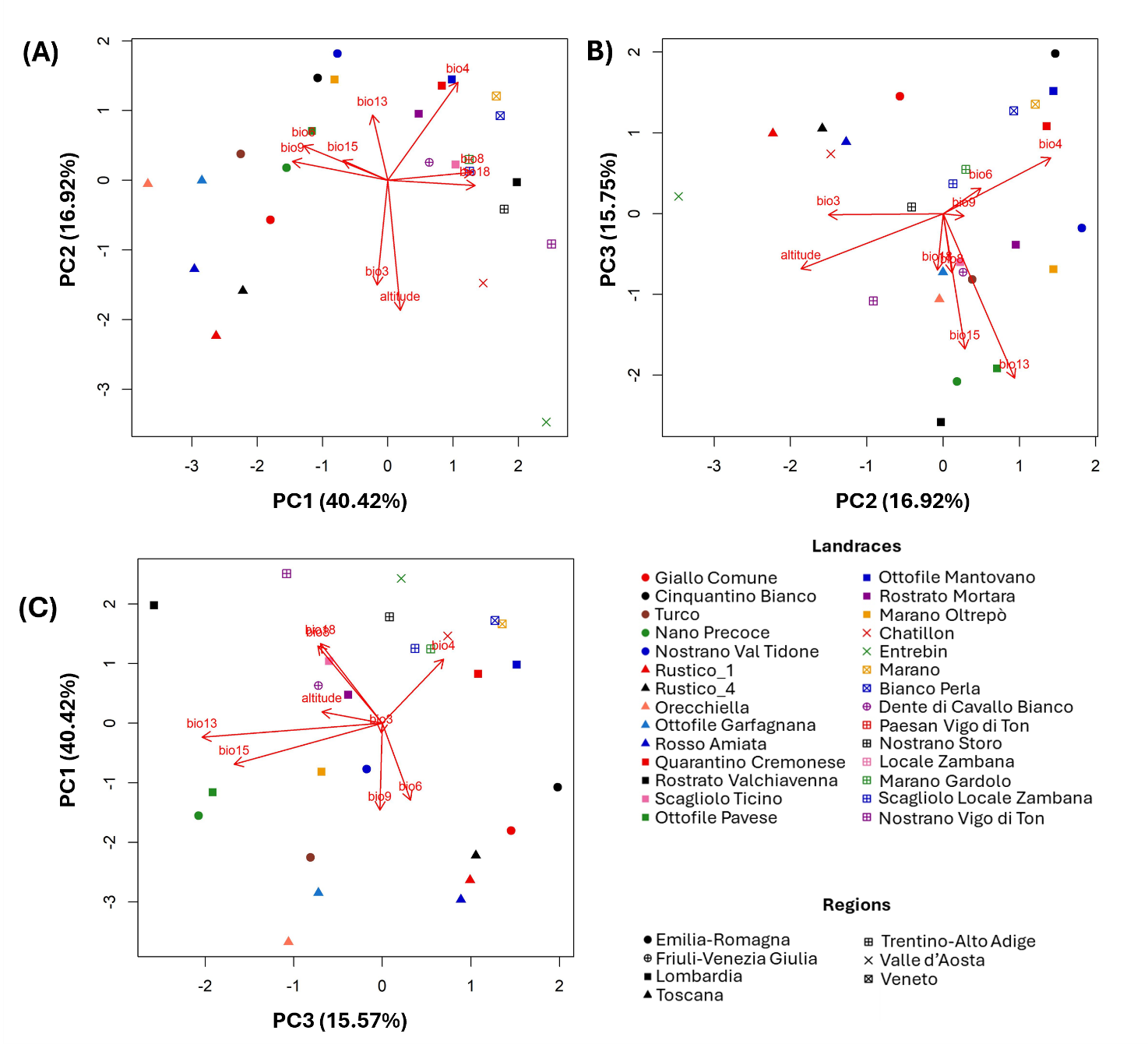


**Figure S11**: Bioclimatic (BIOs) and Moran's Eigenvector Maps (MEMs) predictors explaining genomic variation in Northern-Central Italian landraces. (A) Ranked accuracy importance, in terms of predictive power, of the top bioclimatic and spatial variables, based on gradient forest (GF) analysis. (B) Biplot of the biological space represented by principal components of the transformed grid. The first three principal components were transformed into a defined RGB color palette, where red is defined by values of PC1 + PC2, green by negative values of PC2, and blue by PC3 + PC2 − PC1. Different adaptive environments across the cropping area are displayed, where similar colors represent similar alleles at the predicted loci (r^2^ > 0); in the biplot, axes report the portion of bioclimatic variance (%) explained by the PCs of the transformed grid. (C) GF-transformed bioclimatic variables across the cropping area of maize landrace collection. Colors are based on the biplot of the biological space as in panel (B). Black dots represent landraces’ collection sites.


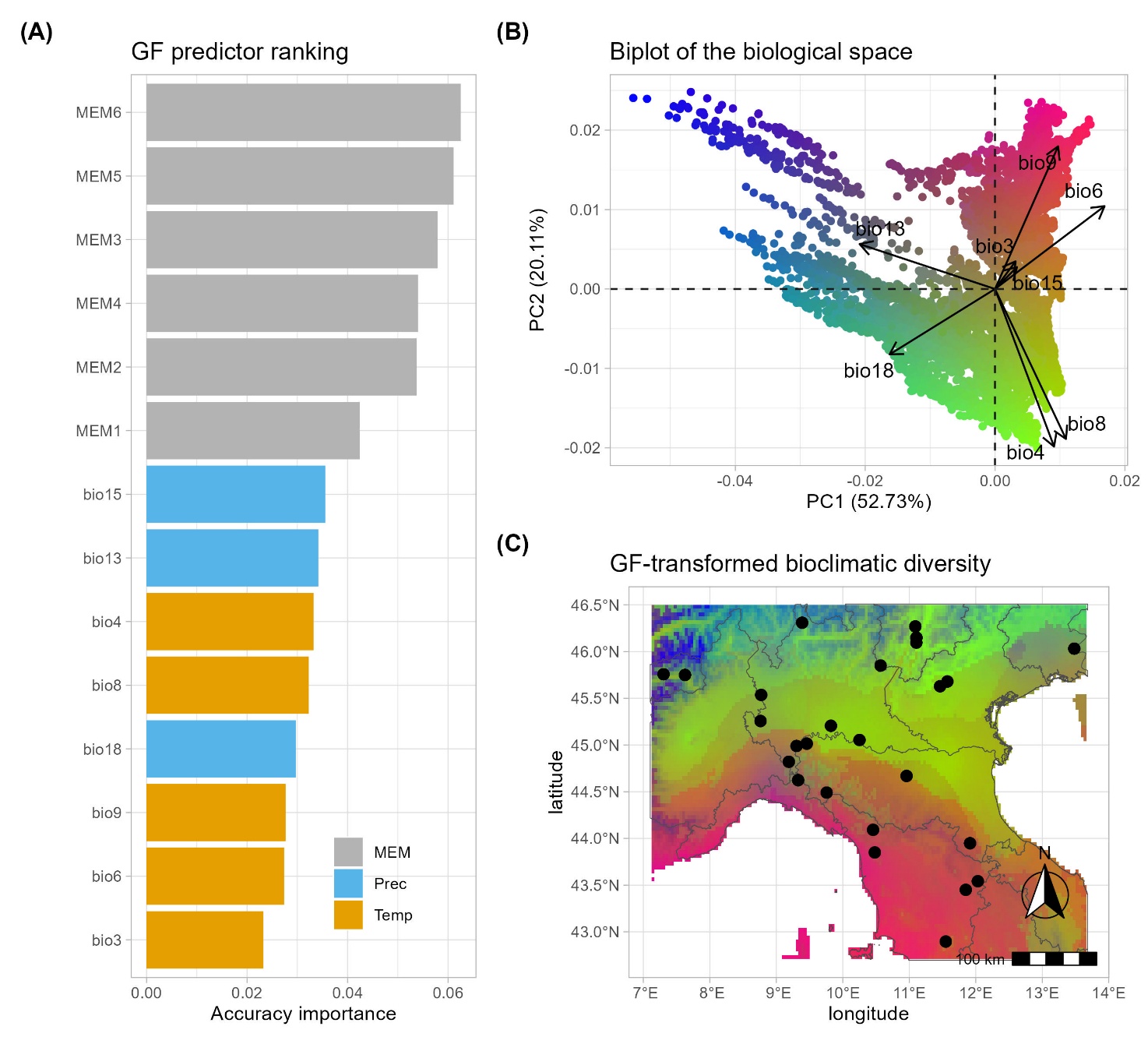

Supplement: Supplementary file 1 — Table S1: Detailed information regarding individual landrace names, precise collection points, and geographic coordinates of the original collection sites of the 28 Italian landraces under study. Germplasm collection was retrieved from Institut Agricole Régional (IAR); Council for Agricultural Research and Economics, Research Centre for Cereal and Industrial Crops (CREA‐CI); Agenzia Regionale per lo Sviluppo e l'Innovazione del Settore Agricolo e Forestale (A.R.S.I.A.); Department of Earth and Environmental Sciences of Università degli Studi di Pavia (UNIPV); Department of Sustainable Crop Production of Università Cattolica del Sacro Cuore (UCSC). Whenever available, references reporting morphological description and additional information were added. Table S2: List of the bioclimatic variables and their respective codes derived from WorldClim (Fick and Hijmans 2017). Table S3: Linkage disequilibrium half‐decay. Figure S1: SNP density plot across the 10 chromosomes of maize representing number of SNPs within 1 Mb window size. The horizontal axis represents the chromosome length in Mb. Different colors correspond to SNP density. Figure S2: Genome‐wide linkage disequilibrium (LD) estimated from 140 maize individuals deriving from the 28 different maize Italian landraces. LD values plotted in a rolling window along each chromosome, with line colors corresponding to the legend. Black arrowheads mark centromere positions (A). LD decay as a function of physical distance between markers, expressed in megabases (Mb) (B). The difference in absolute LD values between the two panels reflects the use of distinct estimation methods: raw rolling‐window calculations in panel A and interpolated values in panel B. Figure S3: Kinship matrix represented as heatmap of pairwise similarities among the 140 individuals deriving from the 28 different maize Italian landraces. Red and blue colors represent low and high similarities, respectively. On the left and top side, a hierarchical tree [file EVA-19-e70186-s001.docx]
